# Cranial shape diversification in horses: variation and covariation patterns under the impact of artificial selection

**DOI:** 10.1186/s12862-021-01907-5

**Published:** 2021-09-21

**Authors:** Pauline Hanot, Jamsranjav Bayarsaikhan, Claude Guintard, Ashleigh Haruda, Enkhbayar Mijiddorj, Renate Schafberg, William Taylor

**Affiliations:** 1 Department of Archaeology, Max Planck Institute for the Science of Human History, Kahlaische Straße 10, 07745 Jena, Germany; 2 National Museum of Mongolia, 1 Juulchin Street, Ulaanbaatar, 15160 Mongolia; 3 Unité d’Anatomie Comparée, Ecole Nationale Vétérinaire de l’Agroalimentaire et de l’Alimentation, Nantes Atlantique - ONIRIS, Route de Gachet, CS 40706, 44307 Nantes Cedex 03, France; 4 Groupe d’Etudes Remodelage osseux et bioMateriaux (GEROM), Unité INSERM 922 LHEA/IRIS-IBS, Université d’Angers, 4 rue Larrey CHU d’Angers, Angers, France; 5 Central Natural Science Collections (ZNS), Martin-Luther University Halle-Wittenberg, Domplatz 4, 06108 Halle (Saale), Germany; 6 School of Archaeology, University of Oxford, 1-2 South Parks Road, Oxford, OX1 3TG UK; 7 Department of Archaeology, Ulaanbaatar State University, Luvsantseveen Street, 5th Khoroo, 15th Khoroolol, Bayanzurkh District, Ulaanbaatar, 13343 Mongolia; 8 University of Colorado-Boulder, Museum of Natural History, Boulder, CO USA

**Keywords:** Artificial selection, Geometric morphometrics, Horse, Morphological integration, Skull

## Abstract

The potential of artificial selection to dramatically impact phenotypic diversity is well known. Large-scale morphological changes in domestic species, emerging over short timescales, offer an accelerated perspective on evolutionary processes. The domestic horse (*Equus caballus*) provides a striking example of rapid evolution, with major changes in morphology and size likely stemming from artificial selection. However, the microevolutionary mechanisms allowing to generate this variation in a short time interval remain little known. Here, we use 3D geometric morphometrics to quantify skull morphological diversity in the horse, and investigate modularity and integration patterns to understand how morphological associations contribute to cranial evolvability in this taxon. We find that changes in the magnitude of cranial integration contribute to the diversification of the skull morphology in horse breeds. Our results demonstrate that a conserved pattern of modularity does not constrain large-scale morphological variations in horses and that artificial selection has impacted mechanisms underlying phenotypic diversity to facilitate rapid shape changes. More broadly, this study demonstrates that studying microevolutionary processes in domestic species produces important insights into extant phenotypic diversity.

**Supplementary Information:**

The online version contains supplementary material available at 10.1186/s12862-021-01907-5.

## Introduction

The phenotypic diversification of domestic species provides a unique and accelerated perspective on evolutionary processes. Artificial selection has proven able to strongly impact the phenotype of domestic taxa over short time frames, producing great amount of morphological disparity often exceeding that of wild counterparts [1–6]. Indeed, sustained selection by breeders (e.g. for specific morphological, functional or behavioral features) can generate novel shape variation and contribute to large-scale phenotypic diversification in a few generations [3]. Among domestic taxa, the morphological diversification in domestic horses (*Equus caballus*) appears as particularly suitable for investigating rapid evolutionary processes having produced substantial shape variation in a few short centuries [7]. Indeed, in terms of both breeding practices and genomic makeup, domestic livestock such as extant horse breeds largely has its origins in the eighteenth century [8–10].

This ability of artificial selection to strongly impact the morphological features of domestic animals raises the issue of the existence of microevolutionary mechanisms facilitating rapid shape changes [11]. Phenotypic diversification is underpinned by several mechanisms which determine the variation that is available for selection to act upon. Notably, the developmental and functional relationships between the different component parts of organisms are known to influence patterns of morphological variation [12–14]. This tendency of morphological traits to covary, or “morphological integration”, is thus a key factor influencing morphological diversification under selection [15–19]. A set of highly correlated morphological traits, acting in a semi-autonomous way, is referred as a module [20]. Morphological modularity and integration are tightly related to evolution as they are thought to influence the “evolvability” (i.e. capacity to evolve) [19] of morphological traits, by constraining the variation of individual traits or facilitating evolution through coordinated shape changes [21–23]. Selective processes may cause changes in modularity patterns or in magnitude of integration, and therefore these can be examined as a way to understand how interactions among traits drive or limit the generation of variation in evolution [15, 19].

Cranial structures are commonly used as a model for studying morphological modularity as they are functionally and developmentally well known, providing thus hypotheses of modular patterning [15, 24, 25]. Previous studies demonstrated the conservation of cranial modularity patterns (i.e. relationships between traits) across placental mammals [24, 26, 27], including equids [4]. Conversely, the magnitude of morphological integration (i.e. intensity of the association between traits) has been shown to vary considerably across taxa, which could have consequences on evolvability [26, 27] and thus facilitate cranial diversification in domestic taxa [1, 3, 6, 28]. Whether modularity constrains or facilitates evolution is still a subject of debate, and no clear relationship between degree of modularity and shape disparity has yet been demonstrated [1, 22, 28]. In the genus *Equus*, a previous study demonstrated a lower magnitude of morphological integration in domestic than in wild taxa. This suggests that artificial selection would be associated with reduced inter-trait relationships, thus potentially contributing to increase flexibility and enhance shape diversification [4]. This could also suggest that variable intensities of alteration in the magnitude of integration could potentially be observed across horses, according to the degree to which they have been submitted to artificial selection.

The wild ancestor of domestic horses no longer exists, and the last surviving population of wild horses is the Przewalski’s horses (*Equus przewalskii*) [29]. It constitutes a distinct species, only other representative of the caballine lineage. Although Przewalski’s horse went “extinct in the wild” in the 1960s [30], they have survived in captivity [31, 32] and, since the late twentieth century, have been progressively reintroduced into the wild [33]. As a different species whose morphology has also likely been impacted by modern captivity, inbreeding depression [29, 34] and potentially human management in the past [35], Przewalski’s horses cannot provide a direct analogue for pre-domesticated horses in studying domestication processes. However, they do nonetheless provide a population of closely-related horses that is not subjected to artificial selection.

The influence of selective breeding may also be assessed within populations of domestic horses, as different evolutionary pathways may explain the formation of different horse breeds. Some current standardized breeds have been subject to high levels of artificial selection and have been forged by reproductive isolation imposed by breeders [36]. This is true of most racehorse breeds, whose breeding is aimed at particular morphological features or athletic performance, and of draft horses, on which considerable selective pressures were exerted, mainly on overall size or body mass [37]. In contrast to breeds formed through deliberate human choice for specific features (e.g. conformation, performance), other breeds might be better characterized as “landraces” (deriving their shared genetic and morphological traits from natural conditions due to long isolation within a specific environment and having been mostly shaped without deliberated breeding decisions) [36]. These horses are generally free-ranging and are breed under conditions of minimal human intervention [38, 39]. Finally, feralization, which is the process by which domestic animals return to the wild, constitutes a third kind of evolutionary pathway. In this case, phenotypic traits of the rewilded animals may have been impacted by natural selection despite their ancestral state of domestication [40–43].

In the present study, we contribute to assess the impact of artificial selection on the cranial morphology of domestic horses using 3D geometric morphometrics. We explore variation in extant groups to determine whether morphological differences between horse populations reflect divergent evolutionary mechanisms implicated in their formation. We also investigate the impact of artificial selection on modularity and integration patterns, to gain insight into underpinning evolutionary mechanisms having allowed the dramatic shape diversification in domestic horses. Hypothesizing that the varying degree of artificial selection to which they have been subject could have differently impacted their morphological traits, we compare the shape variation and covariation patterns among different groups or breeds, known to having been submitted to varying degrees of artificial selection: highly standardized breeds (i.e. race- and draft horses), landraces (i.e. Mongolian, Icelandic, Shetland, Pottok), domestic breeds returned to the wild since several generations (i.e. American feral horses) and last surviving species of wild horses, supposed to have not been subject to artificial selection (i.e. Przewalski’s horses). We explore the shape variation to better understand how artificial selection would have impacted morphological diversification in horses. We then investigate potential differences among these groups in shape covariation. The aim here is to detect potential changes in the structure of modularity, in magnitude or in patterns of integration, and to try to relate them to the varying intensities of artificial selection to which the groups have been submitted.

## Material and methods

### Material

Our analyzed dataset includes a total of 91 skulls from both domestic (*Equus caballus*, n = 74) and Przewalski’s (*Equus przewalskii*, n = 17) horses housed in the collections of several institutions (see Additional file 1). The domestic horses include 21 breeds or landraces, selected to be representative of a large range of diversity in morphology and size: draft (n = 20) and racehorses (n = 21) of various breeds, Mongolian (n = 15), Icelandic (n = 3), Shetland (n = 4), Pottok (n = 3) and American feral horses (n = 8). Due to the small sample size linked to individual breeds, the racing and draft breeds, respectively, were grouped together in most analyses, according the classification of the International Federation for Equestrian Sports, on the basis of functional and genetic criteria. The total sample consists of both males and females. Only adult specimens with all permanent teeth were used (older than 4 years; see Additional file 1).

### Acquisition of data

**Fig. 1.**
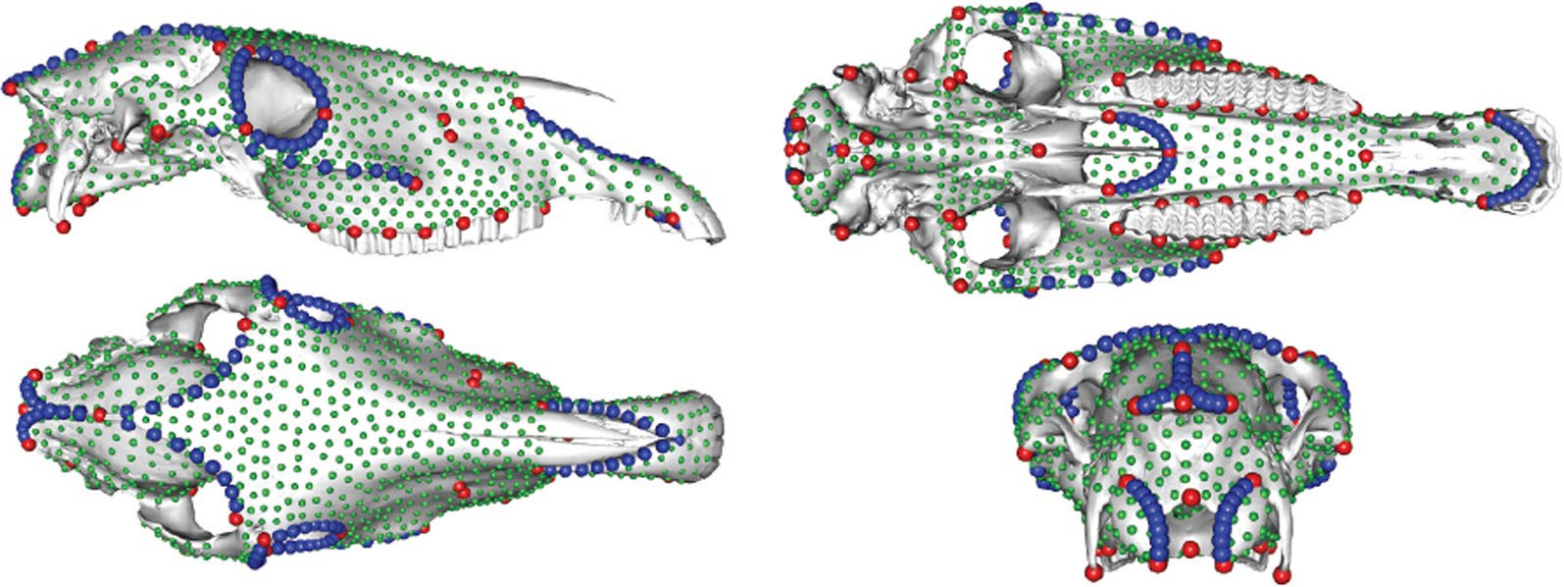
3D view of horse skull showing the location of the 69 anatomical landmarks (in red), 162 sliding semilandmarks placed on curves (in blue) and 1250 surface sliding semilandmarks (in green). See Additional file 2 for landmark definition

Skulls were digitized in three dimensions using several devices (an Artec Space Spider for n = 21, Artec Eva for n = 22, NextEngine laser scanner for n = 22 and photogrammetry for n = 26; see Additional file 1). Bone shape was quantified using a set of anatomical landmarks and sliding semilandmarks on curves and surfaces. We defined a total of 1482 landmarks, including: 69 anatomical landmarks (from Hanot et al. [44]), 162 sliding semilandmarks placed on 20 curves and constrained by anatomical landmarks, and 1250 surface sliding semilandmarks (Fig. 1). Anatomical landmarks and curves were placed on the three-dimensional bone models using the software IDAV Landmark v. 3.0 [45]. We manually digitized surface sliding semilandmarks on a template and then semi-automatically projected these onto each mesh via Thin-Plate Spline (TPS) deformation using the “placePatch” function from the R package *Morpho* [46]. Semilandmarks on curves and surfaces were slid along their tangent vectors/planes to minimize bending energy using the “slider3d” function from *Morpho* package [46]. Symmetrization of the landmark coordinates along the median plan was performed using the “symmetrize” function from *Morpho* package [46].

### Shape analyses

A generalized Procrustes Analysis (GPA) was implemented on the landmark data to remove the effects of location, scale, and orientation of the configurations [47]. We then performed a Principal Component Analysis (PCA) on the Procrustes residuals to reduce the dimensionality of the multivariate datasets [48–50] producing new independent variables (Principal Components, PC) maximizing the variance within the data. The distribution of the data in shape space was displayed by plotting the two first PCs. Visualizations of the shapes associated with extreme parts of the PCs were produced using a TPS deformation of the consensus surface.

We tested differences in shape and size between groups using respectively a Multivariate Analysis of Variance (MANOVA), on PC accounting for more than 95% of the shape variability, and an Analysis of Variance (ANOVA), with Benjamini–Hochberg correction for multiple comparisons [51]. The effect of allometry was assessed by regressing shape against the log_10_-transformed centroid size (CS). To determine if the different groups have a common allometry, we performed a Procrustes ANOVA to test the homogeneity of allometric slopes. Allometry-free shapes were extracted from the residuals of the multivariate regression models [52]. The analyses below were then performed on both normal and allometry-free shape variables.

We assessed the impact of sexual dimorphism on shape by testing the difference between males and females using a MANOVA on shape data accounting for more than 95% of the shape variability. A two-way MANOVA was also used to assess the interaction between sexual dimorphism and difference between groups (of breeds). Due to the small number of male specimens with known castration status in our study sample, we did not test the potential impact of gelding on shape variation.

We performed Canonical Variate Analyses (CVA) on the first PCs (accounting for more than 95% of the shape variability) to describe the differences among groups. Visualizations of the shapes associated with extreme parts of the CVs were produced using a TPS deformation of the consensus surface from a projection of the CVs into the original space.

We computed the Procrustes variance to assess morphological disparity [53] within each of the main groups (racehorses, Mongolian, draft and Przewalski’s horses). To make disparity values comparable across modules, values were scaled by being divided by the number of landmarks and semilandmarks included in each module [54]. Pairwise comparisons between groups were also performed with Benjamini–Hochberg correction for multiple comparisons. We carried out these analyses using the “morphol.disparity” function from the *Geomorph* package [55].

### Integration and modularity

For a better grasp of the processes underlying the generation of variation, we explored shape covariation patterns. The structure of modularity within the skull and its conservation across horses were examined, as well as that of patterns of inter-module morphological integration. Magnitude of between and within-module integration was also assessed, and then, related to module variance to evaluate how integration influences evolvability. Comparisons between the different groups were finally conducted to question the impact of artificial on modularity and integration.

#### Modular patterning

**Fig. 2:**
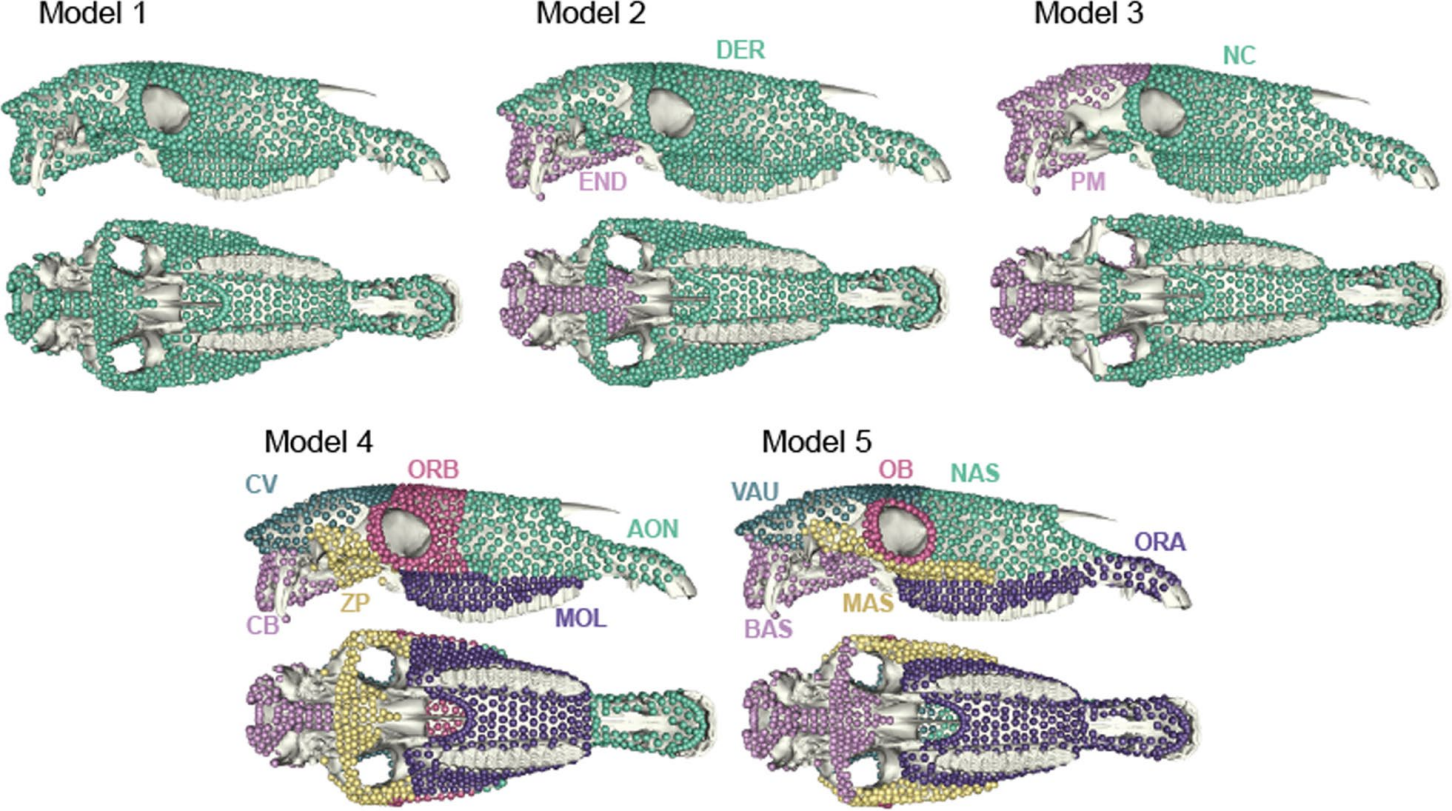
Alternative partitions of the horse skull with model 1: no module; model 2: ossification (*DER* dermal, *END* endochondral); model 3: tissue origin (*NC* neural crest, *PM* paraxial mesoderm); model 4: mammalian (*AON* anterior oral-nasal, *ORB* orbital, *MOL* molar, *CB* basicranium, *ZP* zygomatic-pterygoid, *CV* cranial vault); model 5: functional (*ORA* oral, *NAS* nasal, *OB* orbital, *MAS* masticatory, *BAS* basicranium, *VAU* vault)

We examined the general structure of modularity in the horse skull using hypotheses of functional and developmental influences. Five alternative partitions of landmarks into modules were defined (Fig. 2) according to models previously proposed for skull modularity: (1) absence of distinct modules; (2) ossification model (dermal/endochondral); (3) tissue origin model (neural crest/paraxial mesoderm); (4) mammalian model (anterior oral-nasal/orbital/molar/basicranium/zygomatic-pterygoid/cranial vault; Goswami [24]); (5) functional model (oral/nasal/orbital/masticatory/basicranium/vault; Cheverud [15]). Hypotheses for modularity were investigated with two different approaches: using the EMMLi (“Evaluating Modularity with Maximum Likelihood”) analysis and the Covariance Ratio (CR) [56]. EMMLi is a maximum likelihood approach, implemented in the *EMMLi* package [57], which allows to compare different models of modularity. Because it is not exhaustive in its comparison of models and has been demonstrated as favoring the most-parameterized models [54, 58], we used it coupled with the CR to see if both methods support similar models of modularity. The CR uses the pairwise covariances between variables to quantify modular structure, with modularity in the structure considered as significant when the CR is small relative to the distribution of values obtained under the null hypothesis of random associations of variables. To compute it, we used the “modularity.test” function from *Geomorph* library [55]. This procedure was carried out on each group separately to confirm the hypothesized stability in modular patterning across therian mammals [24]. Because the use of surface semilandmarks may impact modularity patterns (by exaggerating within-module correlations and thus increasing global modularity) [54, 59–61], the procedure was also computed on landmarks and curves only, for matters of comparison. The supported model was then used in further analyses of integration and modularity.

For each module, we performed separate Procrustes superimpositions to examine their shape variation irrespective of their position in the skull [47, 62]. A CVA was conducted on shape data and visualizations of the shape changes were produced using a TPS deformation of the consensus surface. We assessed morphological disparity computing the Procrustes variance [53] with pairwise comparisons between groups.

#### Covariation patterns

To investigate covariation between the different modules, we performed Partial Least Squares (PLS) analyses on the adjacent modules. The two block-PLS (2B-PLS) extracts the principal axes of covariation from a covariance matrix of the two shape datasets [63, 64]. The first PLS axes were plotted and associated shape deformations visualized using a TPS deformation of the consensus surface. We performed 2B-PLS using the “two.b.pls” function from the *Geomorph* package [55]. To compare covariation patterns among groups, we computed the angular difference of the first PLS axis for each group and tested the null hypothesis that the axes are no more similar than vectors having random directions using *MorphoJ* [65].

#### Magnitude of morphological integration

The degree of cranial modularity was assessed using the CR [56]. The CR value and associated effect size (Z-score which provides a standardized measure) [58] describe the degree of modularity within the structure, with low values corresponding to a high degree of modularity. Being insensitive to sample size or number of variables, this measure is well adapted to small sample groups. To compare the effect sizes among groups, we performed two-sample z-tests (with Benjamini–Hochberg correction for multiple comparisons) using the “compare.CR” function from *Geomorph* library [55].

The eigenvalue dispersion of the covariance matrices, computed from standard deviation, was used as a measure of integration within each module [66, 67]. To allow comparison between different matrices, their respective size was taken into account by dividing the observed standard deviation of eigenvalues by the theoretical maximum, producing a relative standard deviation [66, 68]. To obtain a result invariant to sample size, the expected value for the particular sample size (with no integration) was computed to calculate the deviation from the expected value [67]. The range of the relative eigenvalue variance is from zero to one, a value of zero corresponding to an absence of integration (i.e. all eigenvalues being equal). These integration indexes were computed using the “CalcEigenVar” function from the *evolqg* library [69].

Finally, the magnitude of morphological integration between adjacent modules was assessed by 2B-PLS using the “integration.test” function from the *Geomorph* library [55].

For statistical matters, the magnitude of integration was assessed only in the main groups (racehorses, Mongolian, draft and Przewalski’s horses). Moreover, the procedure was computed on landmarks and curves only, for matters of comparison. For all the analyses previously described, we considered test results as significant when p-values (p) were below 0.05. All the plots were performed using the *ggplot2* library [70].

## Results

### Size and shape variation

**Fig. 3:**
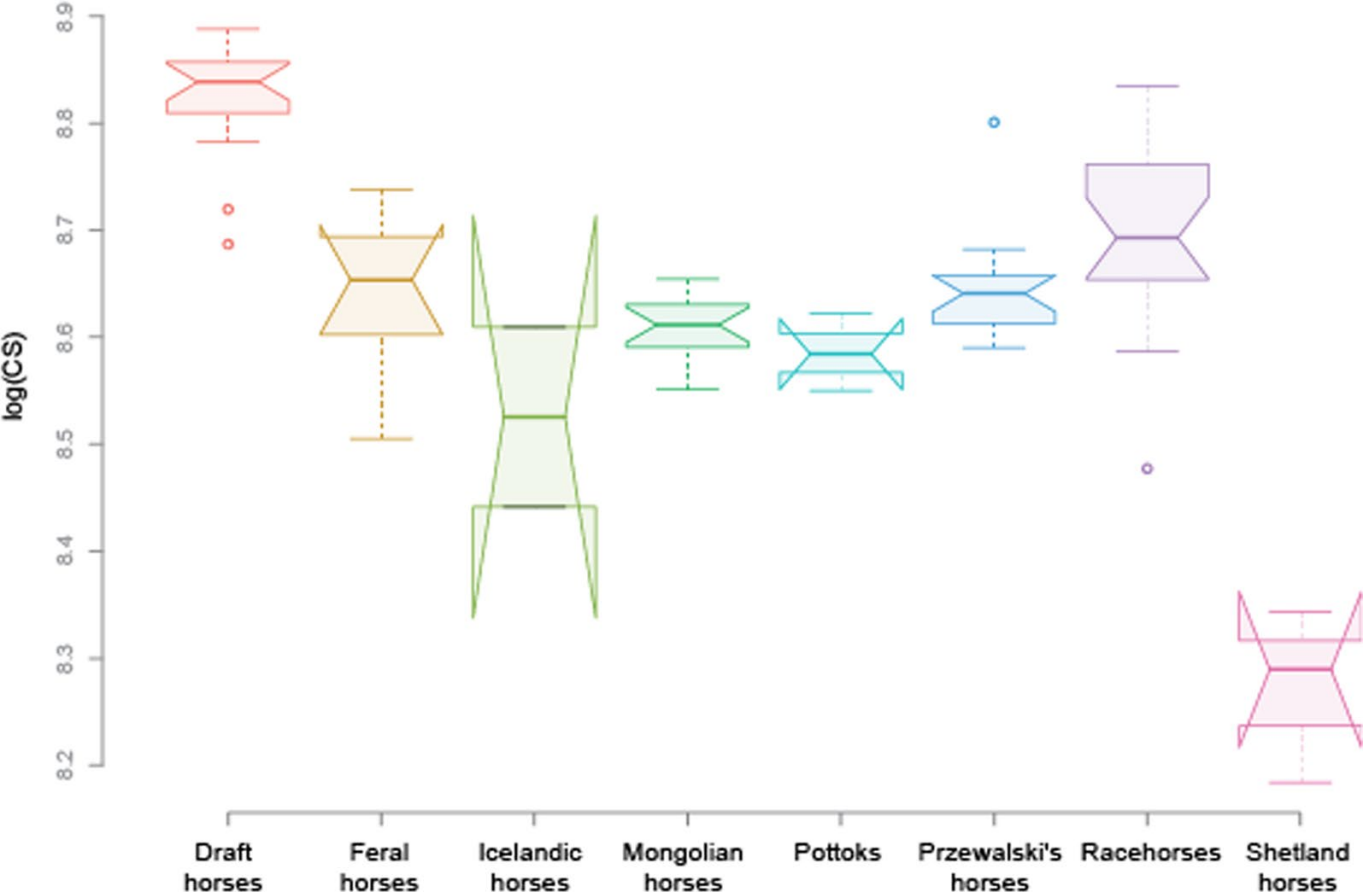
Boxplots of the variation in log-transformed centroid size of the skull for the different groups

MANOVAs on shape data reveal significant pairwise differences among all the main groups (i.e. racehorses, Mongolian horses, draft horses and Przewalski’s horses; p < 0.05) but no significant difference between males and females (p > 0.05). The ANOVA on CS indicates significant pairwise differences between several breed groups (Table 1) with draft horses displaying higher bone CS than all the other groups, racehorses displaying higher bone CS than most of the groups and Shetland horses displaying lower bone CS than all the other groups (Fig. 3).

**Table 1:** Pairwise comparisons of centroid size between groups (significant differences with *; p < 0.05)

	Draft horses	Feral horses	Icelandic horses	Mongolian horses	Pottoks	Przewalski’s horses	Racehorses
Feral horses	3.7e−09*						
Icelandic horses	2.4e−08*	2.9e−02*					
Mongolian horses	1.5e−14*	2.7e−01	9.6e−02				
Pottoks	6.9e−08*	2.1e−01	3.2e−01	5.4e−01			
Przewalski’s horses	1.2e−12*	9.5e−01	2.4e−02*	2.1e−01	2.0e−01		
Racehorses	2.4e−08*	6.1e−02	7.7e−04*	2.9e−04*	9.4e−03*	1.5e−02	
Shetland horses	< 2e−16*	1.3e−12*	6.1e−05*	2.5e−12*	7.0e−08*	9.3e−14*	< 2.0e−16*

The p-values were adjusted using a Benjamini–Hochberg correction

**Fig. 4:**
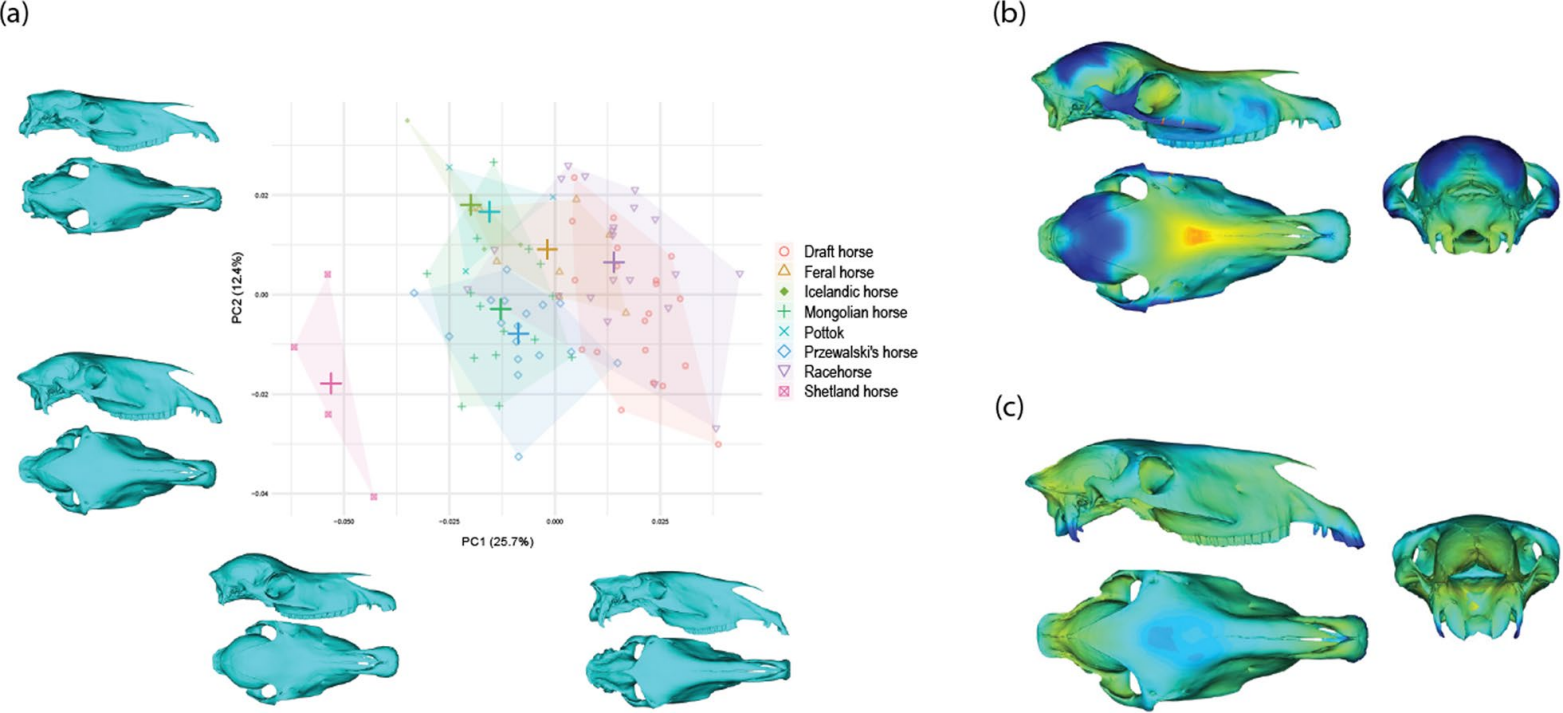
Scatter plot of the two first PCs of the PCA performed on the cranial shape data (crosses represent the centroid value for each group) with visualization of the shape changes along the axes (**a**). Anatomical location and intensity of the shape deformation associated with PC1 (**b**) and PC2 (**c**) using distances from the shapes at the negative to positive extreme of the axis (from blue to red)

The multivariate regression of the shape against the log10-transformed CS indicates significant and strong impact of size on shape (R^2^ = 0.93). This strong influence of allometry on shape variation is reflected by the distribution of the specimens according to size along PC1 (which accounts for 25.7% of the total shape variation; Fig. 4). Anatomically, smaller skulls are broader and characterized by a rounder braincase, a concave nasal bone and larger orbits comparatively to total skull size.

The result of the Procrustes ANOVA shows that the different breeds share a common allometry (p > 0.05). A multivariate regression on the whole sample was thus performed to obtain allometry-free shapes.

### Allometry-free shape variation

The result of the MANOVA on allometry-free shapes indicates pairwise differences among all the main groups (i.e. racehorses, Mongolian horses, draft horses and Przewalski’s horses; p < 0.05). On allometry-free shapes, differences between males and females are significant (p < 0.05; see Additional file 3). However, the two-way MANOVA demonstrates the absence of interaction between breed groups and sexual differences, thus allowing to consider that sexual dimorphism does not bias our between-group results.

**Fig. 5:**
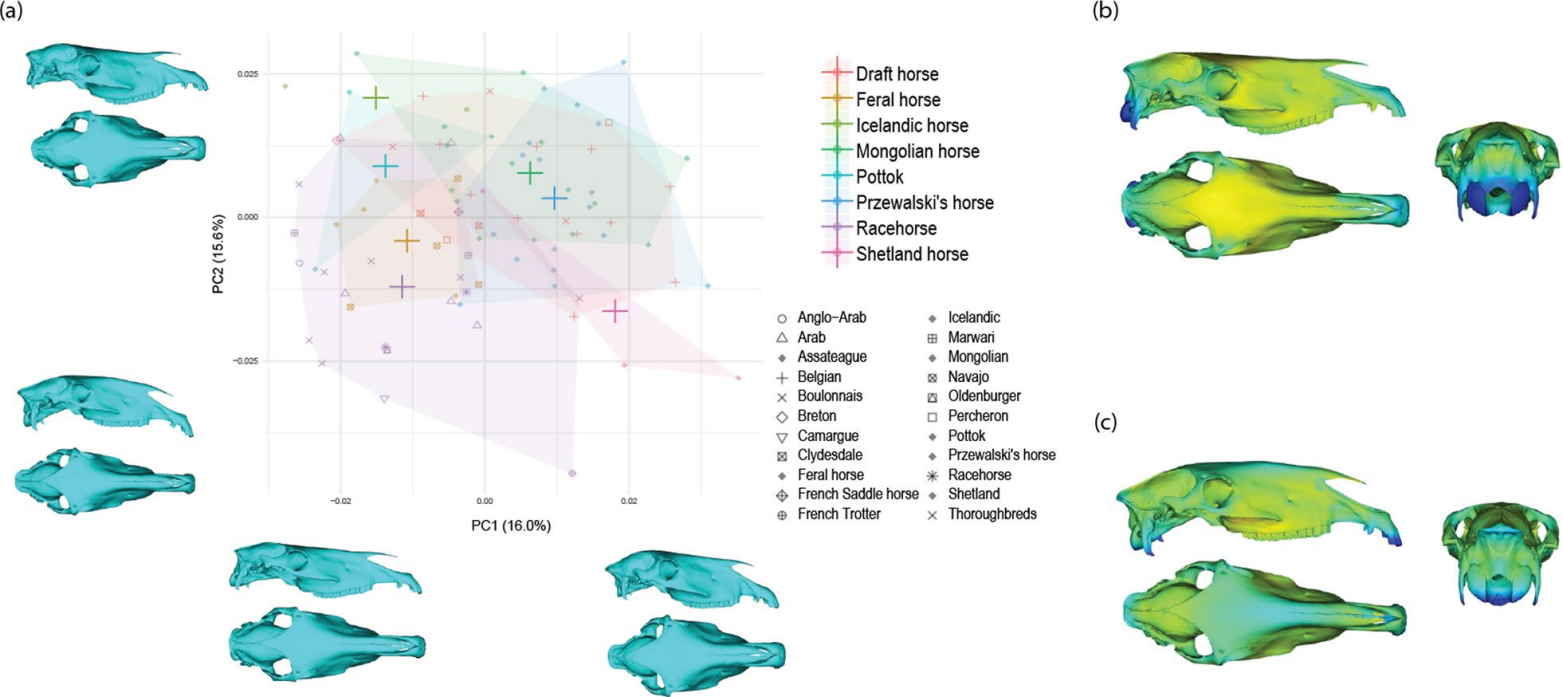
Scatter plot of the two first PCs of the PCA performed on the allometry-free cranial shape data (crosses represent the centroid value for each group) with visualization of the shape changes along the axes (**a**). Anatomical location and intensity of the shape deformation associated with PC1 (**b**) and PC2 (**c**) using distances from the shapes at the negative to positive extreme of the axis (from blue to red)

The distribution of the specimens along the first axes of the PCA on allometry-free shapes does not reveal clear differentiation between individual race and draft horse breed groups, supporting their bundling into larger groups (Fig. 5). Similarly, we observe an important overlap between the main groups. Globally, the two first axes of the PCA express slight differentiation between racehorses and the three other main breed groups (i.e. Przewalski’s, Mongolian and draft horses). Other landraces (i.e. Icelandic, Pottok and Shetland horses) and to a lesser extent, American feral horses, tend to occupy an intermediate position.

**Fig. 6:**
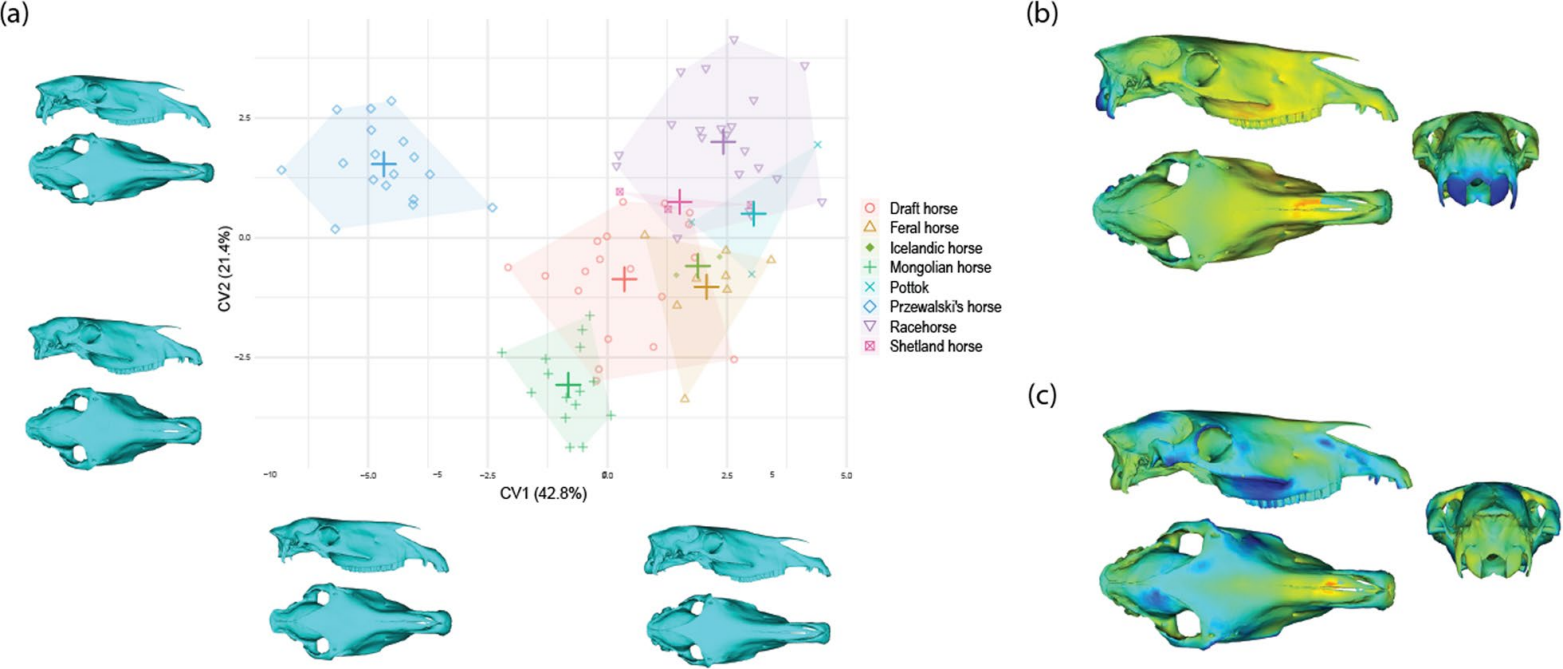
Scatter plot of the two first CVs of the CVA performed on the allometry-free cranial shape data (42 PCs) with visualization of the shape changes along the axes. **a** Anatomical location and intensity of the shape deformation associated with CV1 (**b**) and CV2 (**c**) using distances from the shapes at the negative to positive extreme of the axis (from blue to red)

To simplify the description of differences among breed groups, we performed a CVA on allometry-free shape data. The distribution of specimens along the first CV (55.6%) shows that shape differentiation is mainly driven by the species difference, as the axis clearly distinguishes Przewalski’s horses from domestic ones (Fig. 6). Anatomical changes along CV1 mainly concern the occipital region of the skull: condyles are more developed posteriorly in domestic horses, and exhibit a less extended nuchal crest. This analysis also reveals a difference in the general width of the skull, with Przewalski’s horses displaying a broader skull from incisive to orbital areas. Finally, Przewalski’s horses exhibit a slightly straighter nasal bone and a more robust incisive area.

Domestic horses are differentiated along the second CV (30.6%), with racehorses occupying the positive side of the axis. Shetland ponies, Pottok and Icelandic horses are in an intermediate position, followed by draft and American feral horses. Finally, Mongolian horses occupying the negative extreme. Anatomically, CV2 mainly expresses the variation in the width and height of the skull (from incisive to orbital areas), with negative part of the axis corresponding to broader and higher skulls which present a rounder braincase and less developed occipital condyles. Variation in the shape of the incisive bone can be noticed as well, with its anterio-ventral extension at the positive extreme of the axis.

Comparisons of the Procrustes variance among the main groups show several significant differences. Przewalski’s and racehorses display the highest variances, whereas draft horses appear as the least morphologically variable (Table 2).

**Table 2:** Procrustes variance and p-values obtained from the pairwise comparisons between the main groups (significant differences with *; p < 0.05)

	Draft horses	Mongolian horses	Przewalski’s horses	Racehorses
**Procrustes variance**	**1.07e−03**	**1.21e−03**	**1.62e−03**	**1.45e−03**
Mongolian horses	4.4e−01			
Przewalski’s horses	1.8e−02*	4.2e−02*		
Racehorses	4.2e−02*	2.4e−01	3.7e−01	–

The p-values were adjusted using a Benjamini–Hochberg correction

### Allometry-free shape variation of the modules

Results obtained from the EMMLi approach indicate that the best-supported model of cranial modularity in horses is the Model 4 (mammalian model) [24], with both within-module and between-modules distinct correlation values (see Additional file 4: Table S1). This same model is also supported in each of the main groups independently (see Additional file 4: Tables S2 to S5). In accordance with EMMLi results, the lowest CR value is obtained for the Model 4 (CR = 0.61/p < 0.05; see Additional file 4: Table S7) with an effect size (Z-score = − 22.2) significantly lower than that of other models (see Additional file 4: Table S7). Similar results were obtained from EMMLi analyses computed on curves and landmarks only Additional file 4: Table S6). The lowest CR value for analysis performed on curves and landmarks only is obtained for the Model 4, with a lowest Z-score obtained for the Model 5 but not significantly different from that of the Model 4 (see Additional file 4: Table S7). The Model 4 was thus retained for further analyses.

**Fig. 7:**
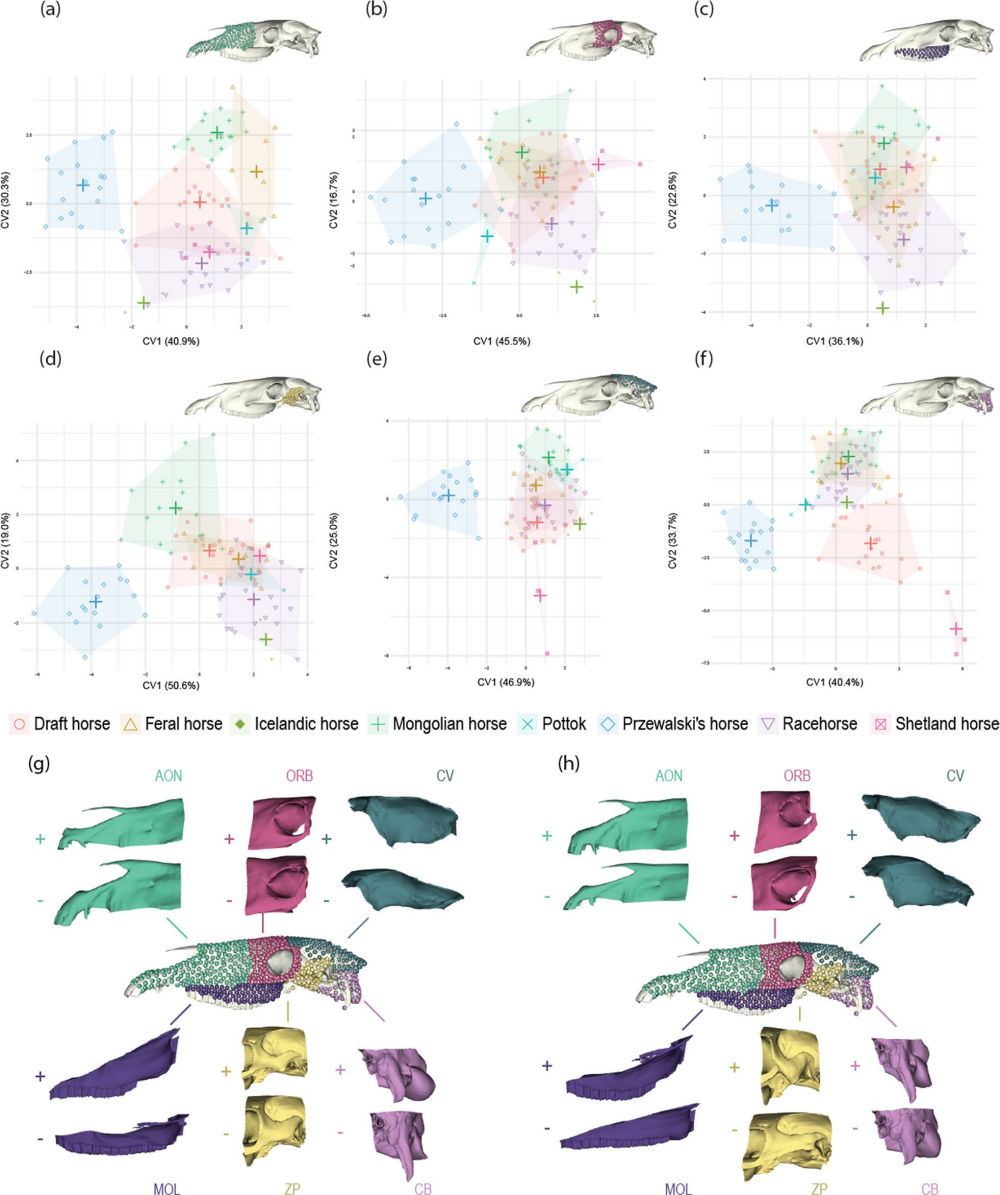
Scatter plots of the two first CVs of the CVA performed for each module (**a** AON; **b** ORB; **c** MOL; **d** ZP; **e** CV; **f** CB) on 95% of the allometry-free shape data (corresponding respectively to 29, 23, 27, 33, 25 and 33 PCs) and visualization of the shape changes along CV1 (**g**) and CV2 (**h**) (+: extreme positive; −: extreme negative). Crosses represent the centroid value for each group. *AON* anterior oral-nasal, *ORB* orbital, *MOL* molar, *CB* basicranium, *ZP* zygomatic-pterygoid, *CV* cranial vault

To describe the shape diversity within modules, CVAs were performed separately on each of the six modules (anterior oral-nasal/AON, orbital/ORB, molar/MOL, basicranium/CB, zygomatic-pterygoid/ZP, cranial vault/CV). For all modules, the first axis distinguishes domestic from Przewalski’s horses (as already observed on the complete skull; Fig. 7). Concerning the second axis, the patterning we observed in the whole-skull dataset is again replicated within the modules AON and ZP (with racehorses occupying one morphological extreme, Mongolian horses occupying the other, and draft horses pooling with feral horses and landraces in an intermediate position). A quite similar pattern can be observed for ORB and MOL, although the groups are less clearly distinguishable. For these four modules, it should however be noted that the two Icelandic horses from our sample pool with racehorses, which differs from the results obtained on the complete skull. The only exceptions to this pattern are the modules CV and CB, for which Shetland ponies occupy the negative extreme of the axis, with draft horses the only other distinguishable group in CB.

Comparisons among modules of the Procrustes variance indicate that CB displays the highest adjusted variance value (3.4e−05) followed by CV (1.4e−05) and ZP (1.1e−05), whereas the lowest variances are obtained for AON (5.6e−06), ORB (6.5e−06) and MOL (7.5e−06).

Comparisons among groups show no significant difference in Procrustes variance for ORB, ZP, CB and CV (Table 3). Concerning other modules, we observe the same tendency found on the complete skull, with the highest variance values obtained in Przewalski’s horses, and to a lesser extent in racehorses, and the lowest variance occurring in draft horses. A unique pattern can be seen in the MOL module, wherein Mongolian horses are the only group to display a variance value as high as that observed in Przewalski’s horses.

**Table 3:** Procrustes variance within modules and p-values obtained from the pairwise comparisons between the main groups (significant differences with *; p < 0.05)

	Draft horses	Mongolian horses	Przewalski’s horses	Racehorses
**AON**				
**Procrustes variance**	**1.60e−03**	**2.05e−03**	**2.51e−03**	**2.48e−03**
Mongolian horses	2.5e−01			
Przewalski’s horses	2.1e−02*	2.5e−01		
Racehorses	1.8e−02*	2.5e−01	9.3e−01	–
**ORB**				
** Procrustes variance**	**1.22e−03**	**1.61e−03**	**1.83e−03**	**1.56e−03**
Mongolian horses	4.5e−01			
Przewalski’s horses	2.2e−01	5.6e−01		
Racehorses	4.5e−01	8.5e−01	5.6e−01	–
**MOL**				
**Procrustes variance**	**1.34e−03**	**2.42e−03**	**3.15e−03**	**2.07e−0**3
Mongolian horses	3.4e−02*			
Przewalski’s horses	6.0e−03*	1.3e−01		
Racehorses	1.3e−01	4.3e−01	3.4e−02*	–
**ZP**				
**Procrustes variance**	**1.47e−03**	**1.55e−03**	**2.37e−03**	**1.85e−03**
Mongolian horses	8.0e−01			
Przewalski’s horses	5.4e−02	6.0e−02		
Racehorses	3.6e−01	4.6e−01	3.0e−01	–
**CB**				
**Procrustes variance**	**3.91e−03**	**3.70e−03**	**4.39e−03**	**7.50e−03**
Mongolian horses	8.3e−01			
Przewalski’s horses	8.2e−01	8.1e−01		
Racehorses	4.5e−01	4.5e−01	6.6e−01	–
**CV**				
**Procrustes variance**	**2.94e−03**	**2.93e−03**	**3.80e−03**	**2.96e−03**
Mongolian horses	1.0			
Przewalski’s horses	5.3e−01	5.3e−01		
Racehorses	1.0	1.0	5.3e−01	–

The p-values were adjusted using a Benjamini–Hochberg correction

*AON* anterior oral-nasal, *ORB* orbital, *MOL* molar, *CB* basicranium, *ZP* zygomatic-pterygoid, *CV* cranial vault

### Modularity and integration

The highest CR effect size (corresponding to lower modular signal) computed on the complete skull was obtained on draft horses (CR = 0.71, effect size = − 9.833), followed by Przewalski’s horses (CR = 0.67, effect size = − 9.835), Mongolian horses (CR = 0.73, effect size = − 9.837) and racehorses (CR = 0.67, effect size = − 9.839). Pairwise comparisons reveal significant differences in the degree of modularity among all the groups. Comparable results are observed on the dataset including landmarks and curves only, with the lowest Z-score obtained for racehorses and the highest one for Przewalski’s horses (see Additional file 5). Concerning the within-module magnitude of integration, the eigenvalue dispersion of the covariance matrix shows the highest degree of morphological integration in CV (cranial vault) and the lowest in CB (basicranium; Table 4). Similar results were also obtained looking at each group separately, except in two cases: in Przewalski’s horses, for which CB is the most highly integrated module, followed by CV; and in Mongolian horses, for which ORB (orbital) and MOL (molar) display a stronger degree of integration than CV. Similar results are obtained when considering landmarks and curves only (see Additional file 5).

**Table 4:** Eigenvalue dispersion of the covariance matrice indicating the degree of morphological integration within each module

	Total sample	Draft horses	Mongolian horses	Przewalski’s horses	Racehorses
AON	0.33	0.33	0.37	0.39	0.35
ORB	0.34	0.36	0.50	0.40	0.37
MOL	0.35	0.37	0.47	0.43	0.39
ZP	0.33	0.39	0.35	0.45	0.33
CB	0.28	0.36	0.35	0.61	0.31
CV	0.44	0.45	0.42	0.54	0.51

*AON* anterior oral-nasal, *ORB* orbital, *MOL* molar, *CB* basicranium, *ZP* zygomatic-pterygoid, *CV* cranial vault

We quantified the magnitude of covariation between adjacent modules using 2B-PLS. Some significant pairwise differences in PLS effect size can be observed, with the strongest degree of integration obtained for the AON/MOL pair, and, to a lesser extent, ZP/CV (Table 5). Similar results are obtained looking at each group separately (see Additional file 6) as well as considering landmarks and curves only (see Additional file 5).

**Table 5:** Pairwise comparisons of the effect sizes of PLS analyses indicating the degree of morphological integration between the adjacent modules (significant differences with *; p < 0.05)

	PLS effect size	AON/MOL	AON/ORB	ORB/MOL	ORB/CV	MOL/ZP	ZP/CV	ZP/CB
AON/MOL	7.49							
AON/ORB	4.71	1.1e−01						
ORB/MOL	6.18	3.5e−01	5.7e−01					
ORB/CV	3.28	9.1e−03*	3.5e−01	9.6e−02				
MOL/ZP	3.24	9.1e−03*	3.4e−01	7.3e−02	9.1e−01			
ZP/CV	7.19	5.7e−01	3.4e−01	6.9e−01	3.2e−02*	3.0e−02*		
ZP/CB	4.01	6.6e−02	7.3e−01	3.5e−01	5.7e−01	5.5e−01	1.9e−01	
CB/CV	3.63	3.2e−02*	6.0e−01	2.6e−01	6.9e−01	6.5e−01	1.1e−01	7.9e−01

The p-values were adjusted using a Benjamini–Hochberg correction

*AON* anterior oral-nasal, *ORB* orbital, *MOL* molar, *CB* basicranium, *ZP* zygomatic-pterygoid, *CV* cranial vault

**Fig. 8:**
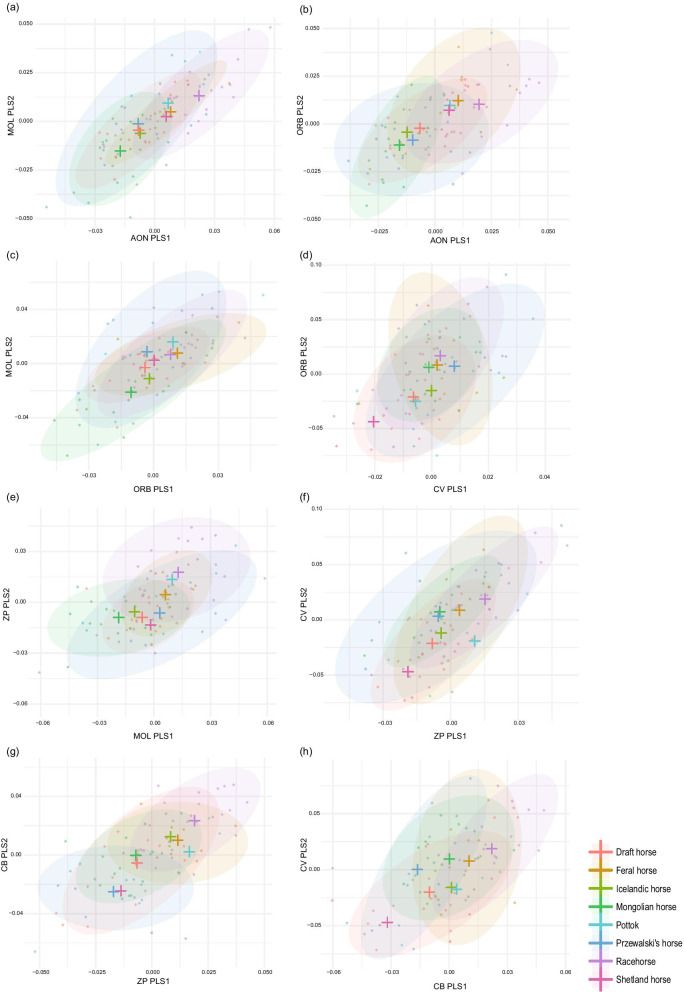
Scatter plot of the first PLS axes describing covariation between adjacent cranial modules. *rPLS* PLS correlation coefficient, *TC* total covariation. Crosses represent the centroid value for each group. *AON* anterior oral-nasal, *ORB* orbital, *MOL* molar, *CB* basicranium, *ZP* zygomatic-pterygoid, *CV* cranial vault

To visualize integration patterns, we produced scatter plots of the first PLS axes describing covariation between the adjacent modules (Fig. 8). For better visibility, 90% data ellipses were drawn [71]. A common trend in the distribution of the specimens stands out in pairs of modules from the anterior part of the skull (AON/MOL, AON/ORB, MOL/ZP and, to a lesser extent, MOL/ORB) with Mongolian and racehorses occupying distinct positions along the PLS axis. Other groups exhibit more intermediate positions but with draft horses tending to pool with Mongolian horses, and feral horses tending to pool with racehorses. Przewalski’s horses generally occupy almost all of the axis. A different tendency can be observed on the first PLS axis between the ZP and CB modules, with racehorses differing from Przewalski’s horses, whereas the other groups occupy intermediate positions. Finally, the distribution of the specimens along the axes of covariation between posterior modules (ORB/CV, ZP/CV, CB/CV) does not show clearly structured patterns.

Angular comparisons between PLS axes reveal no significant differences between groups in most cases, indicating that all the groups share similar covariation patterns in most cases. The only exception concern the Przewalski’s horses, for which the main PLS axis between ORB and CV differs in direction from that of Mongolian horses (88.9°), and for which the main PLS axis between ZP and CB differ from that Mongolian (87.0°) and draft horses (85.8°). The covariation between CV and CB should also be mentioned as the plot reveals the parallel distribution of Przewalski’s and racehorses along the first PLS axis, suggesting different covariation patterns without angular difference.

## Discussion

### Cranial shape variation in the horse: the role of allometry and artificial selection in the morphological diversification

Results from this study first demonstrate that allometry strongly contributes to shape diversification in domestic horses, among which considerable size disparity exists. Allometry accounts for a large part of the shape variation in the horse’s skull, with 93% of the shape changes explained by size. Our approach using surface semilandmarks allows for the precise depiction of these changes, insofar as they are mainly located on regions deprived from anatomical landmarks (e.g. cranial vault, nasal bone). Shetland ponies can be differentiated from the other breeds based on their broad skulls displaying a round braincase, concave face and large orbits. These characteristics, which correspond to features generally described as juvenile [72–74], have been imputed to differences in the ontogenetic trajectory lengths and slopes among horse breeds, producing comparable skull shapes in adult ponies than in taller horses of younger age [73].

When considering the CVA based on the allometry-free shape, species variation produces a clear differentiation between domestic and Przewalski’s horses which appears largely related to the skull width, and to the shape of the occipital bone. Differences in posture and motion of the neck related to activity could contribute to explain why shape changes appear mainly concentrated in the occipital region within our sample of wild and domestic horses. Indeed, traction or riding have for instance been suggested to cause nuchal enthesopathy [75, 76], as well as differences in the captivity conditions which could have notably involved differences in the feed intake posture [76]. These variations being related to individuals’ life history, further studies comparing free-ranging and captive horses are needed to explore this question more deeply, in order to disentangle the impact of artificial selection and the potential effects of captivity on the shape of the occipital bone.

Among domestic horses, the second axis separates racehorses, which experience the strongest degree of artificial selection in our sample, from Mongolian horses, which live free-ranging lives with a lower degree of human intervention in reproduction and management in comparison to other domestic horses. Mongolian horses display broad skulls with relatively weakly developed occipital condyles, as observed in Przewalski’s horses. Importantly, their skull exhibits a round braincase, suggesting a potential impact of the brain size on the cranial vault shape. Domestication is generally considered to have led to brain size reduction in various domestic animals, notably horses [77]. If Mongolian horses have indeed been subjected to a lower degree of artificial selection, this consideration may explain the relatively larger braincases and shape differences among this group. Draft horses occupy an intermediate position on this axis, which could be related to the lower degree of artificial selection they have undergone on specific morphological features than on body size [78]. Interestingly, American feral horses also occupy an intermediate position. Considering that most contemporary American feral horses stem from light racehorse-types (imported by European settlers from the sixteenth century) [79], this result suggests that the return to wild conditions and the relaxation of artificial selection are accompanied by morphological changes. This would help explain historical observations of various feral horse populations worldwide, from Australian brumbies to West Africa and the Americas, especially concerning skull breadth [80–82]. The ability of feral organisms to revert to wild-type features has been observed in several taxa [6, 40–42, 83]. Further research including a larger number of feral populations is now needed to assess the response of domestic captive-bred horses to natural selection and free-roaming lifestyle. The possibility to consider feral populations as proxies of wild-type ancestral populations (no longer existing for domestic horses) could improve our understanding of domestication mechanisms by allowing to observe a potential reversal of the effects of domestication [84–86]. In the case of dogs, for which the wild ancestor is currently also extinct [87, 88], the interest given to dingoes (as a unique model of feralization because having lived isolated from domestic dogs during around 8000 years [89]) exemplifies the growing attention to the topic of feralization in research on domestication.

Although relatively small sample sizes among our individual draft and racehorse breeds compelled us to group individual draft and racehorse breeds together, the fact that we observed no apparent evidence for structuring of breeds within each of these groups suggests that breed definitions are not the most relevant level of morphological division among domestic horses. Having been subject to crossbreeding and refinement crossing to varying extents, horse breeds exhibit a wide range of within-breed homogeneity [90] and at least some of these breeds are arbitrary from a genetic point of view [36].

The shape variation within racehorses, a domestic group known to experience strong degrees of artificial selection, is as large as the intra-species variation observed within Przewalski’s horses. This demonstrates the ability of artificial selection to produce massive shape diversification over relatively short time frames of a few hundred years [3, 6]. We should caution that, although the sample of Przewalski’s horses included in this study was selected to include a diverse range of life history conditions, with specimens from both captivity and free-roaming conditions, the modern-day representatives of this species originate from a single, small group of founders [29, 31, 32] that have been bred in captivity since their extinction in their original range in the 1960’s [30]. Undoubtedly, this recent history has impacted this group’s morphology. Nonetheless, the fact that we observe a similar within-species variation in our sample of Przewalski’s horses than in a single group of domestic horses is in accordance with what is observed at a wider level in equids [4] and other mammalian taxa [1, 3, 91]. This confirms that the strong artificial selection to which domestic horses have been subjected, aiming to produce various specific conformations and functional features, is a major evolutionary force driving shape diversification.

### Cranial modularity and shape diversification

#### Stasis in patterns, changes in magnitude

Because selection may also alter the developmental and functional processes underlying the generation of variation, we examined the organization of horse cranial covariation. Our results support the idea that a conserved modular system does not limit cranial diversification in horses. The best-supported modular patterning of the skull is the same for all the studied groups and corresponds to that brought out in equids [4] and more generally in therian mammals [24]. Moreover, the vector angles between the main PLS axes reveal that covariation is characterized by similar patterns in all the groups. These results are concomitant with those obtained in various mammalian and avian taxa [1, 6, 27] and contribute to highlight the role of stabilizing selection on functional and developmental processes in maintaining the cranial integration structure [27].

Our results also reveal differences in the intensity of cranial integration among groups. The lowest magnitude of integration is obtained for racehorses whereas draft and Przewalski’s horses provide the highest values. This would appear to indicate that the strong degree of artificial selection to which the race breeds have been subjected may have decreased the magnitude of their cranial integration. This result is consistent with that obtained at the genus level which showed lower magnitudes of integration in domestic than in wild equids [4], although not observed more widely in mammalian domesticates [91]. Taken together, these results suggest that the diversification process related to artificial selection increases modular organization in horses. These relaxed covariation constraints may facilitate the shape diversification observed in the racehorses, and in domestic horses in general [4], without the need to disrupt modularity patterns and inter-modules relationships [26, 27, 92, 93].

#### Influence of morphological integration on shape variance

The modular structure of the skull may also foster independent variation of each module, resulting in a differential degree of shape variance and of intra and inter-module integration. In this study, the highest variance values were observed for the modules from the neurocranium (basicranium/CB and cranial vault/CV). The fact that these two modules are equally variable across draft horses, Mongolian horses, Przewalski’s horses, and racehorses confirms that this trend for the neurocranium applies across all studied groups. The lowest value of disparity was observed for the anterior oral-nasal (AON).

How the degree of integration influences morphological evolution, and thus impacts morphological disparity, is a lingering question [22, 26, 28]. In general, there are two opposing hypotheses: (1) that high magnitude of integration restricts the variation of individual traits, resulting in low morphological disparity and decreased evolutionary flexibility; (2) that high magnitude of integration promotes variation through coordinated morphological changes among traits, resulting in high morphological disparity and increased evolutionary flexibility. This study does not show a simple relationship between shape variance and magnitude of morphological integration, as it has been outlined in previous studies [22, 91]. Instead, the two most variable modules in our sample, which display respectively the lowest (CB) and highest (CV) eigenvalue dispersions, suggest that morphological integration would both constrain (CB) and facilitate (CV) morphological changes. This confirms that there is probably no simple rule relating morphological evolution and integration [22].

A different tendency entirely emerges in our sample of Przewalski’s horses, for which CB and CV are both the most variable and integrated modules, results which support the idea that strong integration facilitates variation in this taxon. In contrast, all three less variable modules (AON, ORB/orbital and MOL/molar) are the three least integrated in Przewalski’s horses. In a horse species which is currently not under artificial selection, our results are thus consistent with strong degrees of integration facilitating shape variation, in accordance with previous results obtained on dingoes and dogs [28], but differing from those obtained at a wider level in wild and domestic taxa of the genus *Equus* [4]. To sum up, while our overall results tend to more often support the facilitation hypothesis, discrepancies among our own results, as well as between our findings and those reported in other studies, leave few firm answers as to how the magnitude of integration impacts shape disparity.

Finally, our results also reveal that the three most variable modules (ZP/zygomatic-pterygoid, CB and CV), which are the three most integrated among wild horses, are also those which most poorly covary with other modules (excepting covariation between ZP and CV). This observation suggests that a strong level of modular independence could also be related to high values of shape variance.

### The role of function and development in integration patterns

The six-module model coincides with functional groupings within the equine skull: the anterior oral-nasal (AON) and molar (MOL) parts are the main modules involved in the masticatory apparatus; the zygomatic-pterygoid (ZP) module is involved in mastication by comprising jaw muscle attachments; the orbit (ORB) module houses the visual structures; the cranial vault (CV) module provides protection to the brain; the cranial base (CB) module is both involved in supporting the braincase and constitutes in the attachment point between the skull and the axial skeleton. Consequently, examining the magnitude of within and inter-module integration enables us to investigate the patterns of functional relationships within the horse skull. Developmental processes, in particular the mode of ossification and tissue origin, also contribute to modular patterning by causing covariation among the structures derived from each of these origins [25]. AON, MOL and ORB are all derived from a same tissue origin (neural crest) and mode of ossification (dermal bones), although ORB may also include paraxial mesoderm-derived tissue and bone formed by endochondral ossification. The CB module only encompasses endochondral bones of paraxial mesoderm origin. Finally, the ZP and CV modules are composed of both neural crest and paraxial mesoderm derived bones and both dermal and endochondral bones [94, 95].

Antero-posterior patterning clearly emerges from the cranial shape variation and covariation in our analyzed sample of horses. The three most variable, integrated and independent modules in Przewalski’s horses are the three posterior ones (ZP, CV and CB). The relative independence of these posterior modules is further highlighted by our PLS analyses, which show no clear differentiation along the first PLS axes, but significant differences in covariation patterns (i.e. angular differences for ORB/CV and ZP/CB or parallel trajectories for CV/CB between the main PLS axes).

#### Anterior region

The common developmental origin of AON, MOL and to a lesser extent, ORB (i.e. neural crest-dermal bones), along with the fusion of facial prominences [25], explains strong inter-module relationships between these units, but functional reasons may also influence this pattern. For instance, strong covariation between AON and MOL is consistent with their shared functional role in the masticatory apparatus, as shared muscle attachments and mechanical activities (i.e. mastication) are known to produce covariation among structures [96]. Covariation between these anterior modules is accompanied by low intra-module integration and low shape variability, implying that inter-module integration could constrain the independent shape variation of the modules via developmental and/or functional constraints. This low shape variance observed in AON and MOL is in accordance with the weaker impact of domestication on the facial skeleton in horses than in many other domestic species [77, 97].

Mongolian horses seem to differ from other horse groups concerning the anterior modules with a specific pattern of variation and covariation in the MOL region. Indeed, within-module integration in MOL for Mongolian horses is higher than in CV, which contrasts with the results obtained for the other groups. To some extent, this difference may reflect lower CV integration in Mongolian horses, which, paired with their broader braincase, suggests that relaxed covariation constraints in CV would facilitate potential variations in brain volume. However, our results also reveal for Mongolian horses an unusual high variance value in MOL (paired with an absence of clear shape differentiation from draft and feral horses). In Carnivora, the MOL region has been identified as a strongly integrated and disparate module [22], a pattern linked to strong selective pressures applied on this area of crucial functional importance in a clade with high ecological and dietary diversity. Functional requirements related to mastication for varied diet is thus one potential explanation for the high variance and magnitude of integration in MOL in Mongolian horses.

#### Posterior region

Both developmental and functional processes also seem to be involved in the modular patterning of the posterior part of the horse skull. In this region, inter-module integration is generally low, which could be explained in part by the diversity of developmental origins across modules in this area of the skull. Strong covariation is, however, observed between ZP and CV, which could be due to their similar developmental pathway (these modules are the only ones to encompass bone deriving from both dermal and endochondral origin, and tissue from both neural crest and paraxial mesoderm).

Functional differentiation may also explain the low degree of inter-module integration in the posterior area of the skull. The brain being the largest organ in the skull of most mammals, its growth is an important driver for CV variation [98]. The evolutionary necessity of CV flexibility could thus explain the relative independence of the neurocranial modules. Domestication in horses is often hypothesized to have produced a reduction in brain size via selection for tameness, potentially modulated by changes in neural crest development [77, 97, 99]. Moreover, artificial selection could have deliberately influenced brain regions in some breeds, in relation to specific uses (e.g. for training or dressage). The high variance value we obtained for CV is thus consistent with variation in brain size linked to selective pressures in domestication. It should be noted that historical CV variance is probably underestimated in our analysis due to the absence of surviving undomesticated conspecifics of *E. caballus*. Strong selective pressures may also have impacted the Przewalski’s horse during its near-extinction in the twentieth century. Due to their time in captivity, Przewalski’s horses are considered as having been subject to a 14% decrease in cranial volume which would be similar to that of domestic horses [85, 100].

The CV module also emerges as the most highly integrated in our dataset. Whereas a strong degree of integration also characterizes the CV in the genus *Equus* [4], among mammals as a whole, this area is differentially integrated. The CV is highly integrated in carnivores, while a reduced magnitude of integration in primates [101, 102], may have allowed for the expansion of the brain [24]. Because of the hypothesized link between CV integration and encephalization, further research on a larger sample of feral horses and wild-raised Przewalski’s horses will help clarify the potential impact of free-roaming lifestyle and artificial selective pressures on cranial volume and CV integration.

Highly variable and poorly integrated across domestic horses, CB is the most integrated module in Przewalski’s horses. This finding is consistent with previous observations by Heck et al. [4], who showed that the CB module had the highest degree of integration in wild equids as compared to domesticated horses. A strongly integrated CB is also found more broadly across mammalian taxa [22, 24], but is usually associated with low shape variance [22]. Taken together, our results suggest that the CB module, generally considered as an evolutionarily conserved region [22, 103], has likely been subjected to significant selection pressure in domestic horses and that relaxed covariation constraints may have facilitated morphological diversification. This scenario would explain high CB variance in domestic horses, and the fact that the occipital appears as a main driver of morphological changes across our study groups. The CB module is derived from a single tissue origin (paraxial mesoderm) and is composed of bones using a single mode of ossification (endochondral). As a result, lower integration in this unit is probably caused by functional factors rather than developmental factors. The CB is implicated in two different functions (supporting the brain and connecting the skull to the axial skeleton), and differential selection could have favored functional dissociation of the within-modules traits. As a consequence of domestication and captivity, potential variations in posture [75] or brain size could have occurred, producing increased flexibility in the CB region with relaxed covariation constraints.

## Conclusion

This study elucidated microevolutionary mechanisms underpinning phenotypic diversification of domestic horse breeds under artificial selection. We confirmed the ability of artificial selection to produce large amounts of shape diversity, in comparing domestic breeds to the extant wild form, the Przewalski’s horse. As already shown in several taxa, we also found that this drastic diversification did not rely upon changes in modularity patterns but rather upon variations in the magnitude of integration between morphological features. Our results also reveal that strong degrees of artificial selection are associated with lower intensity of integration, suggesting that increased independence of cranial modules facilitates rapid shape changes. A particularly high degree of autonomy was obtained for modules located in the posterior region of the skull, an area involved in holding brain and connecting the skull to the axial skeleton. Because of the potential variations in brain size and head posture associated with domestication, an enhanced need for flexibility in this anatomical region could explain this result. Further studies focused on reintroduced Przewalski’s horses will help identify evolutionary changes over generations under natural selection and disentangle these from plastic signals related to life conditions. Additional research involving a larger number of feral horse populations is also needed to evaluate how adaptive responses to natural selection impact cranial shape variation and covariations in domestic animals reverted into a wild state. Finally, by revealing morphological and microevolutionary responses of domestic horses to artificial selection, our findings may help better understand the domestication process in horses and other large mammals. Future research involving archaeological material from early domestication contexts could allow to track down morphological changes related to human impact on the horse over time.

## Supplementary Information

**Additional file 1.** List of the specimens.

**Additional file 2.** Landmark definition.

**Additional file 3.** Analysis of sexual dimorphism.

**Additional file 4.** Study of the modularity structure.

**Additional file 5.** Integration and modularity analyses on landmarks and curves only.

**Additional file 6.** Pairwise comparisons of the effect sizes of PLS analyses for each group.
