# Supplementary material for: Cranial shape diversification in horses: variation and covariation patterns under the impact of artificial selection
Source: BMC Ecol Evol. 2021 Sep 21;21:178. doi: 10.1186/s12862-021-01907-5 (PMC8456661; doi:10.1186/s12862-021-01907-5)
Supplement: Supplementary file 1 — Additional file 1. List of the specimens. [file 12862_2021_1907_MOESM1_ESM.docx]

**Additional file 1**

**Table: List of the specimens.
Abbreviations for institutions:** MNHN-Paris-AC, Muséum national d’Histoire naturelle (Comparative Anatomy) – Paris/France; NMNH-Washington, Smithsonian National Museum of Natural History-Washington/USA; IRSNB-Brussels, Institut Royal des Sciences naturelles de Belgique – Brussels/Belgium; MLU/ZNS/H-Halle -Halle, Zentralmagazin Naturwissenschaftlicher Sammlungen der Martín-Luther-Unversität Halle-Wittenberg (Museum für Haustierkunde « Julius Kühn ») - Halle (Saale)/Germany; ONIRIS-Nantes, Ecole Nationale Vétérinaire, Agroalimentaire et de l’Alimentation Nantes-Atlantique (Comparative Anatomy) – Nantes/France; MSB-Albuquerque, Museum of Southwestern Biology-Albuquerque/USA; NMM- Ulaanbaatar, National Museum of Mongolia – Ulaanbaatar/ Mongolia; NNV Clinic-Chinle, Navajo Nation Veterinary Clinic-Chinle/USA; TAKH-Hures-la-Parade, Association Takh pour le cheval de Przewalski- Hures-la-Parade/France; PACEA, UMR 5199 PACEA Université de Bordeaux-Bordeaux/France.

**Abbreviations for sex:** F, female; M, male; G, gelding.

| **Location** | **Inventory no** | **Species** | **Classification** | **Breed** | **Sex** | **Age (y)** | **Method of digitisation** |
| --- | --- | --- | --- | --- | --- | --- | --- |
| MNHN-Paris-AC (France) | MNHN-ZM-AC-1891-107 | *Equus caballus* | Draft horse | Boulonnais | M | 14 | Artec Space Spider |
| MNHN-Paris-AC (France) | MNHN-ZM-AC-1930-27 | *Equus caballus* | Draft horse | Boulonnais | G | - | Photogrammetry |
| MNHN-Paris-AC (France) | MNHN-ZM-AC-1930-32 | *Equus caballus* | Draft horse | Boulonnais | - | - | Photogrammetry |
| MNHN-Paris-AC (France) | MNHN-ZM-AC-1930-28 | *Equus caballus* | Draft horse | Belgian | - | - | Photogrammetry |
| MNHN-Paris-AC (France) | MNHN-ZM-AC-1930-29 | *Equus caballus* | Draft horse | Belgian | - | - | Photogrammetry |
| MNHN-Paris-AC (France) | MNHN-ZM-AC-1930-30 | *Equus caballus* | Draft horse | Belgian | - | - | Photogrammetry |
| MNHN-Paris-AC (France) | MNHN-ZM-AC-1930-31 | *Equus caballus* | Draft horse | Breton | - | - | Photogrammetry |
| NMNH-Washington (USA) | NMNH-174960 | *Equus caballus* | Draft horse | Percheron | M | - | NextEngine |
| MNHN-Paris-AC (France) | MNHN-ZM-AC-1977-84 | *Equus caballus* | Draft horse | Percheron | M | - | Photogrammetry |
| IRSNB-Brussels (Belgium) | IRSNB-10507 | *Equus caballus* | Draft horse | Belgian | M | - | Artec Space Spider |
| IRSNB-Brussels (Belgium) | IRSNB-12143 | *Equus caballus* | Draft horse | Belgian | M | - | Artec Space Spider |
| IRSNB-Brussels (Belgium) | IRSNB-12144 | *Equus caballus* | Draft horse | Belgian | F | - | Artec Space Spider |
| MLU/ZNS/H-Halle (Germany) | E blg 1 | *Equus caballus* | Draft horse | Belgian | F | - | Artec Eva |
| MLU/ZNS/H-Halle (Germany) | E blg 2 | *Equus caballus* | Draft horse | Belgian | F | 15-18 | Artec Eva |
| MLU/ZNS/H-Halle (Germany) | E blg 3 | *Equus caballus* | Draft horse | Belgian | G | 16 | Artec Eva |
| MLU/ZNS/H-Halle (Germany) | E blg 5 | *Equus caballus* | Draft horse | Belgian | G | 7-8 | Artec Eva |
| MLU/ZNS/H-Halle (Germany) | E blg 6 | *Equus caballus* | Draft horse | Belgian | M | 18 | Artec Eva |
| MLU/ZNS/H-Halle (Germany) | E blg 7 | *Equus caballus* | Draft horse | Belgian | M | 17 | Artec Eva |
| MLU/ZNS/H-Halle (Germany) | E cldd 4 | *Equus caballus* | Draft horse | Clydesdale | F | - | Artec Eva |
| MLU/ZNS/H-Halle (Germany) | E cldd 5 | *Equus caballus* | Draft horse | Clydesdale | F | 18 | Artec Eva |
| MNHN-Paris-AC (France) | MNHN-ZM-AC-1926-124 | *Equus caballus* | Racehorse | Thoroughbreds | - | - | Photogrammetry |
| MLU/ZNS/H-Halle (Germany) | E arb 5 | *Equus caballus* | Racehorse | Thoroughbreds | M | - | Artec Eva |
| MLU/ZNS/H-Halle (Germany) | E arb 6 | *Equus caballus* | Racehorse | Thoroughbreds | M | - | Artec Eva |
| MLU/ZNS/H-Halle (Germany) | E arb 7 | *Equus caballus* | Racehorse | Thoroughbreds | M | - | Artec Eva |
| MLU/ZNS/H-Halle (Germany) | E arb 8 | *Equus caballus* | Racehorse | Thoroughbreds | M | 23 | Artec Eva |
| NMNH-Washington (USA) | NMNH-270900 | *Equus caballus* | Racehorse | Thoroughbreds | M | 35 | NextEngine |
| MNHN-Paris-AC (France) | MNHN-ZM-AC-1927-235 | *Equus caballus* | Racehorse | Arab | M | - | Photogrammetry |
| IRSNB-Brussels (Belgium) | IRSNB-3975 | *Equus caballus* | Racehorse | Arab | M | - | Artec Space Spider |
| MLU/ZNS/H-Halle (Germany) | E arb 2 | *Equus caballus* | Racehorse | Arab | F | 14 | Artec Eva |
| MLU/ZNS/H-Halle (Germany) | E arb 4 | *Equus caballus* | Racehorse | Arab | M | - | Artec Eva |
| MLU/ZNS/H-Halle (Germany) | E arb 1 | *Equus caballus* | Racehorse | Arab | M | - | Artec Eva |
| ONIRIS-Nantes (France) | ONIRIS-CV12 | *Equus caballus* | Racehorse | Anglo-Arab | F | 29 | Photogrammetry |
| MSB-Albuquerque (USA) | MSB-56502 | *Equus caballus* | Racehorse | Quarterhorse | M | - | NextEngine |
| IRSNB-Brussels (Belgium) | IRSNB-13071 | *Equus caballus* | Racehorse | Camargue | F | - | Artec Space Spider |
| ONIRIS-Nantes (France) | ONIRIS-CV10 | *Equus caballus* | Racehorse | French Trotter | M | 21 | Photogrammetry |
| ONIRIS-Nantes (France) | ONIRIS-CV11 | *Equus caballus* | Racehorse | French Saddle horse | F | 25 | Photogrammetry |
| MLU/ZNS/H-Halle (Germany) | E oldb 1 | *Equus caballus* | Racehorse | Oldenburger | F | - | Artec Eva |
| MLU/ZNS/H-Halle (Germany) | E ind 1 | *Equus caballus* | Racehorse | Marwari | F | - | Artec Eva |
| MLU/ZNS/H-Halle (Germany) | E ind 2 | *Equus caballus* | Racehorse | Marwari | M | - | Artec Eva |
| IRSNB-Brussels (Belgium) | IRSNB-1211 | *Equus caballus* | Racehorse | Racehorse | - | - | Artec Space Spider |
| IRSNB-Brussels (Belgium) | IRSNB-12147 | *Equus caballus* | Racehorse | Racehorse | M | - | Artec Space Spider |
| MNHN-Paris-AC (France) | MNHN-ZM-AC-1977-776 | *Equus caballus* | Mongolian horse | Mongolian | - | - | Photogrammetry |
| NMM-Ulaanbaatar (Mongolia) | NMM 1 | *Equus caballus* | Mongolian horse | Mongolian | F | - | NextEngine |
| NMM-Ulaanbaatar (Mongolia) | NMM 2 | *Equus caballus* | Mongolian horse | Mongolian | M | - | NextEngine |
| NMM-Ulaanbaatar (Mongolia) | NMM 4 | *Equus caballus* | Mongolian horse | Mongolian | M | - | NextEngine |
| NMM-Ulaanbaatar (Mongolia) | NMM 5 | *Equus caballus* | Mongolian horse | Mongolian | F | - | NextEngine |
| NMM-Ulaanbaatar (Mongolia) | NMM 6 | *Equus caballus* | Mongolian horse | Mongolian | M | - | NextEngine |
| NMM-Ulaanbaatar (Mongolia) | NMM 7 | *Equus caballus* | Mongolian horse | Mongolian | M | - | NextEngine |
| NMM-Ulaanbaatar (Mongolia) | NMM 9 | *Equus caballus* | Mongolian horse | Mongolian | F | - | NextEngine |
| NMM-Ulaanbaatar (Mongolia) | NMM 10 | *Equus caballus* | Mongolian horse | Mongolian | F | - | NextEngine |
| NMM-Ulaanbaatar (Mongolia) | NMM 15 | *Equus caballus* | Mongolian horse | Mongolian | M | - | NextEngine |
| NMM-Ulaanbaatar (Mongolia) | NMM 16 | *Equus caballus* | Mongolian horse | Mongolian | M | - | NextEngine |
| NMM-Ulaanbaatar (Mongolia) | NMM 17 | *Equus caballus* | Mongolian horse | Mongolian | M | - | NextEngine |
| MLU/ZNS/H-Halle (Germany) | E mgl 1 | *Equus caballus* | Mongolian horse | Mongolian | F | 12 | Artec Eva |
| MLU/ZNS/H-Halle (Germany) | E mgl 2 | *Equus caballus* | Mongolian horse | Mongolian | F | - | Artec Eva |
| MLU/ZNS/H-Halle (Germany) | E mgl 4 | *Equus caballus* | Mongolian horse | Mongolian | F | 13 | Artec Eva |
| MNHN-Paris-AC (France) | MNHN-ZM-AC-1873-385 | *Equus caballus* | Icelandic horse | Icelandic | F | - | Photogrammetry |
| MNHN-Paris-AC (France) | MNHN-ZM-AC-1880-233 | *Equus caballus* | Icelandic horse | Icelandic | - | - | Photogrammetry |
| MNHN-Paris-AC (France) | MNHN-ZM-AC-1975-98 | *Equus caballus* | Icelandic horse | Icelandic | M | 35 | Artec Space Spider |
| MNHN-Paris-AC (France) | MNHN-ZM-AC-1937-59 | *Equus caballus* | Shetland horse | Shetland | M | - | Artec Space Spider |
| MNHN-Paris-AC (France) | MNHN-ZM-AC-1950-8 | *Equus caballus* | Shetland horse | Shetland | - | - | Photogrammetry |
| IRSNB-Brussels (Belgium) | IRSNB-13097 | *Equus caballus* | Shetland horse | Shetland | F | - | Artec Space Spider |
| ONIRIS-Nantes (France) | ONIRIS-CV13 | *Equus caballus* | Shetland horse | Shetland | M | 10 | Photogrammetry |
| IRSNB-Brussels (Belgium) | IRSNB-14209 | *Equus caballus* | Pottok | Pottok | - | - | Artec Space Spider |
| IRSNB-Brussels (Belgium) | IRSNB-16958 | *Equus caballus* | Pottok | Pottok | F | - | Artec Space Spider |
| IRSNB-Brussels (Belgium) | IRSNB-16959 | *Equus caballus* | Pottok | Pottok | F | - | Artec Space Spider |
| MSB-Albuquerque (USA) | MSB-146537.pts | *Equus caballus* | Feral horse | Feral horse | - | - | NextEngine |
| NMNH-Washington (USA) | NMNH-268938 | *Equus caballus* | Feral horse | Assateague | - | - | NextEngine |
| NMNH-Washington (USA) | NMNH-395180 | *Equus caballus* | Feral horse | Assateague | - | - | NextEngine |
| NMNH-Washington (USA) | NMNH-395432 | *Equus caballus* | Feral horse | Assateague | - | - | NextEngine |
| NNV Clinic-Chinle (USA) | NNV-1 | *Equus caballus* | Feral horse | Navajo | - | - | NextEngine |
| NNV Clinic-Chinle (USA) | NNV-2 | *Equus caballus* | Feral horse | Navajo | - | - | NextEngine |
| NNV Clinic-Chinle (USA) | NNV-3 | *Equus caballus* | Feral horse | Navajo | - | - | NextEngine |
| NNV Clinic-Chinle (USA) | NNV-5 | *Equus caballus* | Feral horse | Navajo | - | - | NextEngine |
| MNHN-Paris-AC (France) | MNHN-ZM-AC-1935-486 | *Equus przewalskii* | Przewalski's horse | - | - | 21 | Artec Space Spider |
| MNHN-Paris-AC (France) | MNHN-ZM-AC-1973-109 | *Equus przewalskii* | Przewalski's horse | - | - | - | Artec Space Spider |
| MNHN-Paris-AC (France) | MNHN-ZM-AC-1975-125 | *Equus przewalskii* | Przewalski's horse | - | - | - | Artec Space Spider |
| MNHN-Paris-AC (France) | MNHN-ZM-AC-1977-55 | *Equus przewalskii* | Przewalski's horse | - | - | - | Photogrammetry |
| MNHN-Paris-AC (France) | MNHN-ZM-AC-1980-29 | *Equus przewalskii* | Przewalski's horse | - | - | 5 | Artec Space Spider |
| MNHN-Paris-AC (France) | MNHN-ZM-AC-2000-312 | *Equus przewalskii* | Przewalski's horse | - | - | 25 | Photogrammetry |
| TAKH-Hures-la-Parade (France) | TAKH-AM | *Equus przewalskii* | Przewalski's horse | - | - | 5 | Photogrammetry |
| TAKH-Hures-la-Parade (France) | TAKH-BH | *Equus przewalskii* | Przewalski's horse | - | - | 15 | Photogrammetry |
| IRSNB-Brussels (Belgium) | IRSNB-14207 | *Equus przewalskii* | Przewalski's horse | - | - | - | Artec Space Spider |
| IRSNB-Brussels (Belgium) | IRSNB-17353 | *Equus przewalskii* | Przewalski's horse | - | - | - | Artec Space Spider |
| TAKH-Hures-la-Parade (France) | TAKH-EO | *Equus przewalskii* | Przewalski's horse | - | - | 13 | Photogrammetry |
| MLU/ZNS/H-Halle (Germany) | E wld 1 | *Equus przewalskii* | Przewalski's horse | - | - | - | Artec Eva |
| PACEA (France) | PACEA-M505 | *Equus przewalskii* | Przewalski's horse | - | - | - | Artec Space Spider |
| TAKH-Hures-la-Parade (France) | TAKH-PR | *Equus przewalskii* | Przewalski's horse | - | - | 24 | Photogrammetry |
| TAKH-Hures-la-Parade (France) | TAKH-SA | *Equus przewalskii* | Przewalski's horse | - | - | 19 | Photogrammetry |
| TAKH-Hures-la-Parade (France) | TAKH-SK | *Equus przewalskii* | Przewalski's horse | - | - | 5 | Photogrammetry |
| TAKH-Hures-la-Parade (France) | TAKH-SL | *Equus przewalskii* | Przewalski's horse | - | - | 21 | Photogrammetry |
