# Supplementary material for: Cranial shape diversification in horses: variation and covariation patterns under the impact of artificial selection
Source: BMC Ecol Evol. 2021 Sep 21;21:178. doi: 10.1186/s12862-021-01907-5 (PMC8456661; doi:10.1186/s12862-021-01907-5)
Supplement: Supplementary file 2 — Additional file 2. Landmark definition. [file 12862_2021_1907_MOESM2_ESM.docx]

**Additional file 2**

**Table: Landmark definition**

| Landmark | Definition |
| --- | --- |
| 1 | Most posterior point of the right nasal cavity |
| 2 | Most posterior point of the left nasal cavity |
| 3 | Most dorsal point of the right infraorbital hole |
| 4 | Most dorsal point of the left infraorbital hole |
| 5 | Most ventral point of the right infraorbital hole |
| 6 | Most ventral point of the left infraorbital hole |
| 7 | Most anterior point of the right facial tubercle |
| 8 | Most anterior point of the left facial tubercle |
| 9 | Most anterior point of the right orbit |
| 10 | Most anterior point of the left orbit |
| 11 | Most dorsal point of the right orbit |
| 12 | Most dorsal point of the left orbit |
| 13 | Most posterior point of the right orbit |
| 14 | Most posterior point of the left orbit |
| 15-21 | Most ventro-lateral points of dental alveolus of each right molar tooth |
| 22-28 | Most ventro -lateral points of dental alveolus of each left molar tooth |
| 29-33 | Most ventro -medial points of dental alveolus of each right molar tooth |
| 34-38 | Most ventro -medial points of dental alveolus of each left molar tooth |
| 39 | Most posterior point of the median palatine suture |
| 40 | Point of maximum curvature of the posterior border of the vomer |
| 41 | Most lateral point of the right choana |
| 42 | Most lateral point of the left choana |
| 43 | Most ventral point of the right jugular process |
| 44 | Most ventral point of the left jugular process |
| 45 | Most antero-ventral point of the right occipital condyle |
| 46 | Most antero-ventral point of the left occipital condyle |
| 47 | Position of the basion |
| 48 | Most posterior point of the right occipital condyle |
| 49 | Most posterior point of the left occipital condyle |
| 50 | Most dorsal point of the foramen magnum |
| 51 | Most dorsal point of the right occipital condyle |
| 52 | Most dorsal point of the left occipital condyle |
| 53 | Most dorsal point of the external occipital protuberance |
| 54 | Most proximo-lateral of the right part of the nuchal crest |
| 55 | Most proximo-lateral of the left part of the nuchal crest |
| 56 | Most posterior point of the right part of the alveolar arch of incisors |
| 57 | Most posterior point of the left part of the alveolar arch of incisors |
| 58 | Most anterior point of the external sagittal crest |
| 59 | Most lateral point of the right articular tubercle of the temporal bone |
| 60 | Most lateral point of the left articular tubercle of the temporal bone |
| 61 | Most ventral point of the right retro-articular process |
| 62 | Most ventral point of the left retro-articular process |
| 63 | Most medial point of the right articular tubercle of the temporal bone |
| 64 | Most medial point of the left articular tubercle of the temporal bone |
| 65 | Most anterior point of contact between the right articular tubercle of the temporal bone and the sphenoid bone |
| 66 | Most anterior point of contact between the left articular tubercle of the temporal bone and the sphenoid bone |
| 67 | Most anterior point of the rightt temporal line |
| 68 | Most anterior point of the left temporal line |
| 69 | Most anterior point of the median palatine suture |
