# Supplementary material for: Cranial shape diversification in horses: variation and covariation patterns under the impact of artificial selection
Source: BMC Ecol Evol. 2021 Sep 21;21:178. doi: 10.1186/s12862-021-01907-5 (PMC8456661; doi:10.1186/s12862-021-01907-5)
Supplement: Supplementary file 3 — Additional file 3. Analysis of sexual dimorphism. [file 12862_2021_1907_MOESM3_ESM.docx]

**Additional file 3**


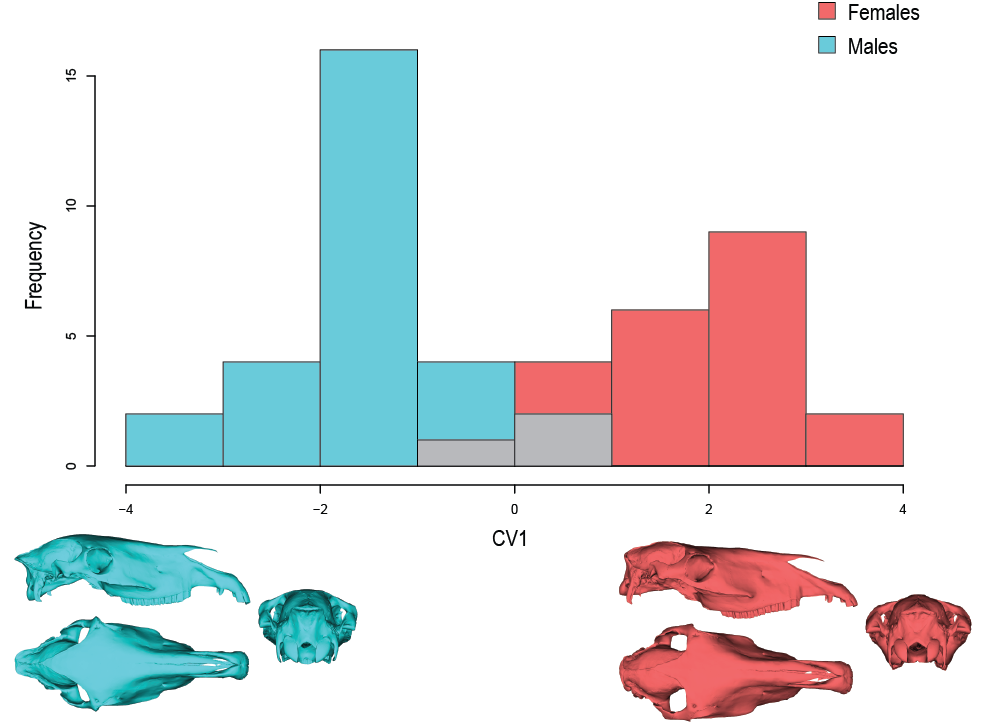


**Figure: Histogram of CV scores from the Canonical Variate Analysis performed on allometry-free shape data (accounting for more than 95% of the shape variability) discriminating males and females and associated shape deformation. The template used for the shape deformation was created from a male specimen (IRSNB-3975), which explains the occurrence of canine teeth on the shape visualizations.**
