# Supplementary material for: Cranial shape diversification in horses: variation and covariation patterns under the impact of artificial selection
Source: BMC Ecol Evol. 2021 Sep 21;21:178. doi: 10.1186/s12862-021-01907-5 (PMC8456661; doi:10.1186/s12862-021-01907-5)
Supplement: Supplementary file 4 — Additional file 4. Study of the modularity structure. [file 12862_2021_1907_MOESM4_ESM.docx]

**Additional file 4**

**Table S1: Results obtained, on the total sample, from the maximum likelihood approach conducted on different models of cranial modularity (EMMLi) indicating the best supported model of modularity (in bold). Include the maximum likelihood values, number of estimated parameters (K), Akaike Information Criterion (AICc), difference between each model’s AICc score and the lowest score observed among models (dAICc), model log-likelihood and posterior probability. The number of between-trait correlations to calculate the likelihood score is 1097421.**

|  |  |  |  |  |  |  |  |  |
| --- | --- | --- | --- | --- | --- | --- | --- | --- |
| **Model** | **Within-module correlation** | **Between-module correlation** | **Maximum likelihood** | **K** | **AICc** | **dAICc** | **Model log-likelihood** | **Posterior probability** |
| No module | - | - | -290058.00 | 2 | 580120.0 | 1115007.9 | 0 | 0 |
| Functional | same | same | 91379.57 | 3 | -182753.1 | 352134.7 | 0 | 0 |
| Functional | separate | separate | 158805.84 | 22 | -317567.7 | 217320.2 | 0 | 0 |
| Functional | separate | same | 111272.86 | 8 | -222529.7 | 312358.2 | 0 | 0 |
| Functional | same | separate | 138912.55 | 17 | -277791.1 | 257096.8 | 0 | 0 |
| Mammalian | same | same | 85500.91 | 4 | -170993.8 | 363894.1 | 0 | 0 |
| **Mammalian** | **separate** | **separate** | **267466.94** | **23** | **-534887.9** | **0.0** | **1** | **1** |
| Mammalian | separate | same | 195214.28 | 9 | -390410.6 | 144477.3 | 0 | 0 |
| Mammalian | same | separate | 157753.57 | 18 | -315471.1 | 219416.7 | 0 | 0 |
| Tissue origin | same | same | -178232.40 | 4 | 356472.8 | 891360.7 | 0 | 0 |
| Tissue origin | separate | separate | -120849.65 | 5 | 241709.3 | 776597.2 | 0 | 0 |
| Tissue origin | separate | same | -120849.65 | 5 | 241709.3 | 776597.2 | 0 | 0 |
| Tissue origin | same | separate | -178232.40 | 4 | 356472.8 | 891360.7 | 0 | 0 |
| Ossification | same | same | -271105.94 | 3 | 542217.9 | 1077105.8 | 0 | 0 |
| Ossification | separate | same | -155697.53 | 4 | 311403.1 | 846290.9 | 0 | 0 |

**Table S2: Results obtained, on the sample of draft horses, from the maximum likelihood approach conducted on different models of cranial modularity (EMMLi) indicating the best supported model of modularity (in bold). The number of between-trait correlations to calculate the likelihood score is 1097421.**

| **Model** | **Within-module correlation** | **Between-module correlation** | **Maximum likelihood** | **K** | **AICc** | **dAICc** | **Model log-likelihood** | **Posterior probability** |
| --- | --- | --- | --- | --- | --- | --- | --- | --- |
| No module | - | - | 699373.9 | 2 | -1398744 | 56288.510 | 0 | 0 |
| Functional | same | same | 714238.4 | 3 | -1428471 | 26561.487 | 0 | 0 |
| Functional | separate | separate | 721291.9 | 22 | -1442540 | 12492.450 | 0 | 0 |
| Functional | separate | same | 717943.1 | 8 | -1435870 | 19161.972 | 0 | 0 |
| Functional | same | separate | 717587.1 | 17 | -1435140 | 19891.965 | 0 | 0 |
| Mammalian | same | same | 717612.8 | 4 | -1435218 | 19814.744 | 0 | 0 |
| **Mammalian** | **separate** | **separate** | **727539.1** | **23** | **-1455032** | **0.000** | **1** | **1** |
| Mammalian | separate | same | 723425.9 | 9 | -1446834 | 8198.508 | 0 | 0 |
| Mammalian | same | separate | 721726.0 | 18 | -1443416 | 11616.236 | 0 | 0 |
| Tissue origin | same | same | 702015.2 | 4 | -1404022 | 51009.815 | 0 | 0 |
| Tissue origin | separate | separate | 706493.0 | 5 | -1412976 | 42056.222 | 0 | 0 |
| Tissue origin | separate | same | 706493.0 | 5 | -1412976 | 42056.222 | 0 | 0 |
| Tissue origin | same | separate | 702015.2 | 4 | -1404022 | 51009.815 | 0 | 0 |
| Ossification | same | same | 699428.0 | 3 | -1398850 | 56182.249 | 0 | 0 |
| Ossification | separate | same | 706150.9 | 4 | -1412294 | 42738.509 | 0 | 0 |

**Table S3: Results obtained, on the sample of racehorses, from the maximum likelihood approach conducted on different models of cranial modularity (EMMLi) indicating the best supported model of modularity (in bold). The number of between-trait correlations to calculate the likelihood score is 1097421.**

| **Model** | **Within-module correlation** | **Between-module correlation** | **Maximum likelihood** | **K** | **AICc** | **dAICc** | **Model log-likelihood** | **Posterior probability** |
| --- | --- | --- | --- | --- | --- | --- | --- | --- |
| No module | - | - | 935491.3 | 2 | -1870979 | 268744.81 | 0 | 0 |
| Functional | same | same | 1017536.7 | 3 | -2035067 | 104656.04 | 0 | 0 |
| Functional | separate | separate | 1039180.8 | 22 | -2078318 | 61405.84 | 0 | 0 |
| Functional | separate | same | 1021443.1 | 8 | -2042870 | 96853.17 | 0 | 0 |
| Functional | same | separate | 1035274.3 | 17 | -2070515 | 69208.71 | 0 | 0 |
| Mammalian | same | same | 1023519.5 | 4 | -2047031 | 92692.47 | 0 | 0 |
| **Mammalian** | **separate** | **separate** | **1069884.7** | **23** | **-2139723** | **0.00** | **1** | **1** |
| Mammalian | separate | same | 1045803.5 | 9 | -2091589 | 48134.47 | 0 | 0 |
| Mammalian | same | separate | 1047600.7 | 18 | -2095165 | 44558.00 | 0 | 0 |
| Tissue origin | same | same | 964136.3 | 4 | -1928265 | 211458.70 | 0 | 0 |
| Tissue origin | separate | separate | 995669.5 | 5 | -1991329 | 148394.35 | 0 | 0 |
| Tissue origin | separate | same | 995669.5 | 5 | -1991329 | 148394.35 | 0 | 0 |
| Tissue origin | same | separate | 964136.3 | 4 | -1928265 | 211458.70 | 0 | 0 |
| Ossification | same | same | 935880.8 | 3 | -1871756 | 267967.71 | 0 | 0 |
| Ossification | separate | same | 958921.8 | 4 | -1917836 | 221887.72 | 0 | 0 |

**Table S4: Results obtained, on the sample of Mongolian horses, from the maximum likelihood approach conducted on different models of cranial modularity (EMMLi) indicating the best supported model of modularity (in bold). The number of between-trait correlations to calculate the likelihood score is 1097421.**

| **Model** | **Within-module correlation** | **Between-module correlation** | **Maximum likelihood** | **K** | **AICc** | **dAICc** | **Model log-likelihood** | **Posterior probability** |
| --- | --- | --- | --- | --- | --- | --- | --- | --- |
| No module | - | - | 947108.7 | 2 | -1894213 | 140801.69 | 0 | 0 |
| Functional | same | same | 980196.3 | 3 | -1960387 | 74628.49 | 0 | 0 |
| Functional | separate | separate | 1000326.5 | 22 | -2000609 | 34405.99 | 0 | 0 |
| Functional | separate | same | 990504.4 | 8 | -1980993 | 54022.28 | 0 | 0 |
| Functional | same | separate | 990018.4 | 17 | -1980003 | 55012.20 | 0 | 0 |
| Mammalian | same | same | 984256.7 | 4 | -1968505 | 66509.51 | 0 | 0 |
| **Mammalian** | **separate** | **separate** | **1017530.5** | **23** | **-2035015** | **0.00** | **1** | **1** |
| Mammalian | separate | same | 1005356.6 | 9 | -2010695 | 24319.87 | 0 | 0 |
| Mammalian | same | separate | 996430.7 | 18 | -1992825 | 42189.64 | 0 | 0 |
| Tissue origin | same | same | 953682.7 | 4 | -1907357 | 127657.58 | 0 | 0 |
| Tissue origin | separate | separate | 968841.0 | 5 | -1937672 | 97343.04 | 0 | 0 |
| Tissue origin | separate | same | 968841.0 | 5 | -1937672 | 97343.04 | 0 | 0 |
| Tissue origin | same | separate | 953682.7 | 4 | -1907357 | 127657.58 | 0 | 0 |
| Ossification | same | same | 947113.4 | 3 | -1894221 | 140794.15 | 0 | 0 |
| Ossification | separate | same | 964466.7 | 4 | -1928925 | 106089.62 | 0 | 0 |

**Table S5: Results obtained, on the sample of feral horses, from the maximum likelihood approach conducted on different models of cranial modularity (EMMLi) indicating the best supported model of modularity (in bold). The number of between-trait correlations to calculate the likelihood score is 1097421.**

| **Model** | **Within-module correlation** | **Between-module correlation** | **Maximum likelihood** | **K** | **AICc** | **dAICc** | **Model log-likelihood** | **Posterior probability** |
| --- | --- | --- | --- | --- | --- | --- | --- | --- |
| No module | - | - | 699373.9 | 2 | -1398744 | 56288.510 | 0 | 0 |
| Functional | same | same | 714238.4 | 3 | -1428471 | 26561.487 | 0 | 0 |
| Functional | separate | separate | 721291.9 | 22 | -1442540 | 12492.450 | 0 | 0 |
| Functional | separate | same | 717943.1 | 8 | -1435870 | 19161.972 | 0 | 0 |
| Functional | same | separate | 717587.1 | 17 | -1435140 | 19891.965 | 0 | 0 |
| Mammalian | same | same | 717612.8 | 4 | -1435218 | 19814.744 | 0 | 0 |
| **Mammalian** | **separate** | **separate** | **727539.1** | **23** | **-1455032** | **0.000** | **1** | **1** |
| Mammalian | separate | same | 723425.9 | 9 | -1446834 | 8198.508 | 0 | 0 |
| Mammalian | same | separate | 721726.0 | 18 | -1443416 | 11616.236 | 0 | 0 |
| Tissue origin | same | same | 702015.2 | 4 | -1404022 | 51009.815 | 0 | 0 |
| Tissue origin | separate | separate | 706493.0 | 5 | -1412976 | 42056.222 | 0 | 0 |
| Tissue origin | separate | same | 706493.0 | 5 | -1412976 | 42056.222 | 0 | 0 |
| Tissue origin | same | separate | 702015.2 | 4 | -1404022 | 51009.815 | 0 | 0 |
| Ossification | same | same | 699428.0 | 3 | -1398850 | 56182.249 | 0 | 0 |
| Ossification | separate | same | 706150.9 | 4 | -1412294 | 42738.509 | 0 | 0 |

**Table S6: Results obtained on the total sample, for curves and landmarks only, from the maximum likelihood approach conducted on different models of cranial modularity (EMMLi) indicating the best supported model of modularity (in bold). The number of between-trait correlations to calculate the likelihood score is 26565.**

| **Model** | **Within-module correlation** | **Between-module correlation** | **Maximum likelihood** | **K** | **AICc** | **dAICc** | **Model log-likelihood** | **Posterior probability** |
| --- | --- | --- | --- | --- | --- | --- | --- | --- |
| No module | - | - | -35681.139 | 2 | 71366.28 | 57865.074 | 0 | 0 |
| Functional | same | same | -16685.213 | 3 | 33376.43 | 19875.223 | 0 | 0 |
| Functional | separate | separate | -13344.672 | 22 | 26733.38 | 13232.178 | 0 | 0 |
| Functional | separate | same | -15317.870 | 8 | 30651.75 | 17150.541 | 0 | 0 |
| Functional | same | separate | -14712.015 | 17 | 29458.05 | 15956.849 | 0 | 0 |
| Mammalian | same | same | -12554.949 | 4 | 25117.90 | 11616.695 | 0 | 0 |
| **Mammalian** | **separate** | **separate** | **-6727.582** | **23** | **13501.20** | **0.000** | **1** | **1** |
| Mammalian | separate | same | -8521.737 | 9 | 17061.48 | 3560.276 | 0 | 0 |
| Mammalian | same | separate | -10760.794 | 18 | 21557.61 | 8056.408 | 0 | 0 |
| Tissue origin | same | same | -33083.858 | 4 | 66175.72 | 52674.514 | 0 | 0 |
| Tissue origin | separate | separate | -30512.160 | 5 | 61034.32 | 47533.117 | 0 | 0 |
| Tissue origin | separate | same | -30512.160 | 5 | 61034.32 | 47533.117 | 0 | 0 |
| Tissue origin | same | separate | -33083.858 | 4 | 66175.72 | 52674.514 | 0 | 0 |
| Ossification | same | same | -35146.581 | 3 | 70299.16 | 56797.958 | 0 | 0 |
| Ossification | separate | same | -28258.748 | 4 | 56525.50 | 43024.292 | 0 | 0 |

**Table S7: Covariance Ratios and associated Z-scores obtained from the different models of cranial modularity**

|  | **Model 2** | **Model 2 (landmarks & curves only)** | **Model 3** | **Model 3 (landmarks & curves only)** | **Model 4** | **Model 4 (landmarks & curves only)** | **Model 5** | **Model 5 (landmarks & curves only)** |
| --- | --- | --- | --- | --- | --- | --- | --- | --- |
| **CR** | 0,72 | 0,68 | 0,69 | 0,66 | 0,61 | 0,49 | 0,62 | 0,53 |
| **Z-scores** | -21,7 | -12,9 | -22,1 | -17,1 | -22,2 | -16,7 | -22,1 | -17,7 |
