# Supplementary material for: Cranial shape diversification in horses: variation and covariation patterns under the impact of artificial selection
Source: BMC Ecol Evol. 2021 Sep 21;21:178. doi: 10.1186/s12862-021-01907-5 (PMC8456661; doi:10.1186/s12862-021-01907-5)
Supplement: Supplementary file 5 — Additional file 5. Integration and modularity analyses on landmarks and curves only. [file 12862_2021_1907_MOESM5_ESM.docx]

**Additional file 5**

**Table S1: Covariance Ratios and associated Z-scores obtained on the complete skull for the different groups, from the dataset including landmarks and curves only.**

|  | **Draft horses** | **Mongolian horses** | **Przewalski's horses** | **Racehorses** |
| --- | --- | --- | --- | --- |
| **CR** | 0.77 | 0.77 | 0.71 | 0.72 |
| **Z-scores** | -13.7 | -14.4 | -13.1 | -14.5 |

**Table S2: Eigenvalue dispersion of the covariance matrice, from the dataset including landmarks and curves only, indicating the degree of morphological integration within each module. AON: anterior oral-nasal, ORB: orbital, MOL: molar, CB: basicranium, ZP: zygomatic-pterygoid, CV: cranial vault.**

|  | **Total sample** | **Draft horses** | **Mongolian horses** | **Przewalski's horses** | **Racehorses** |
| --- | --- | --- | --- | --- | --- |
| **AON** | 0,36 | 0,31 | 0,39 | 0,33 | 0,37 |
| **ORB** | 0,41 | 0,39 | 0,61 | 0,4 | 0,43 |
| **MOL** | 0,42 | 0,36 | 0,44 | 0,47 | 0,48 |
| **ZP** | 0,38 | 0,34 | 0,38 | 0,51 | 0,37 |
| **CB** | 0,38 | 0,33 | 0,41 | 0,77 | 0,38 |
| **CV** | 0,52 | 0,57 | 0,39 | 0,52 | 0,58 |

**Table S3: Pairwise comparisons of the effect sizes of PLS analyses indicating the degree of morphological integration between the adjacent modules, from the dataset including landmarks and curves only. AON: anterior oral-nasal, ORB: orbital, MOL: molar, CB: basicranium, ZP: zygomatic-pterygoid, CV: cranial vault.**

|  | **PLS effect size** | **AON/MOL** | **AON/ORB** | **ORB/MOL** | **ORB/CV** | **MOL/ZP** | **ZP/CV** | **ZP/CB** |
| --- | --- | --- | --- | --- | --- | --- | --- | --- |
| **AON/MOL** | 3.58 |  |  |  |  |  |  |  |
| **AON/ORB** | 2.03 | > 0.05 |  |  |  |  |  |  |
| **ORB/MOL** | 3.02 | > 0.05 | > 0.05 |  |  |  |  |  |
| **ORB/CV** | 2.63 | > 0.05 | > 0.05 | > 0.05 |  |  |  |  |
| **MOL/ZP** | 1.07 | > 0.05 | > 0.05 | > 0.05 | > 0.05 |  |  |  |
| **ZP/CV** | 3.60 | > 0.05 | > 0.05 | > 0.05 | > 0.05 | > 0.05 |  |  |
| **ZP/CB** | 0.50 | > 0.05 | > 0.05 | > 0.05 | > 0.05 | > 0.05 | **< 0.05** |  |
| **CB/CV** | -0.29 | **< 0.05** | > 0.05 | **< 0.05** | **< 0.05** | > 0.05 | **< 0.05** | > 0.05 |
