# Supplementary material for: Cranial shape diversification in horses: variation and covariation patterns under the impact of artificial selection
Source: BMC Ecol Evol. 2021 Sep 21;21:178. doi: 10.1186/s12862-021-01907-5 (PMC8456661; doi:10.1186/s12862-021-01907-5)
Supplement: Supplementary file 6 — Additional file 6. Pairwise comparisons of the effect sizes of PLS analyses for each group. [file 12862_2021_1907_MOESM6_ESM.docx]

**Additional file 6**

**Table: Pairwise comparisons of the effect sizes of PLS analyses indicating the degree of morphological integration between the adjacent modules**

|  |  | **PLS effect size** | **AON/MOL** | **AON/ORB** | **ORB/MOL** | **ORB/CV** | **MOL/ZP** | **ZP/CV** | **ZP/CB** |
| --- | --- | --- | --- | --- | --- | --- | --- | --- | --- |
| **Draft horses** | **AON/MOL** | 3.18 |  |  |  |  |  |  |  |
|  | **AON/ORB** | 2.48 | > 0.05 |  |  |  |  |  |  |
|  | **ORB/MOL** | 3.37 | > 0.05 | > 0.05 |  |  |  |  |  |
|  | **ORB/CV** | 0.92 | > 0.05 | > 0.05 | > 0.05 |  |  |  |  |
|  | **MOL/ZP** | 2.13 | > 0.05 | > 0.05 | > 0.05 |  |  |  |  |
|  | **ZP/CV** | 2.10 | > 0.05 | > 0.05 | > 0.05 | > 0.05 | > 0.05 |  |  |
|  | **ZP/CB** | 2.08 | > 0.05 | > 0.05 | > 0.05 | > 0.05 | > 0.05 | > 0.05 |  |
|  | **CB/CV** | -0.22 | **< 0.05** | > 0.05 | **< 0.05** | > 0.05 | > 0.05 | > 0.05 | > 0.05 |
|  |  | **PLS effect size** | **AON/MOL** | **AON/ORB** | **ORB/MOL** | **ORB/CV** | **MOL/ZP** | **ZP/CV** | **ZP/CB** |
| **Racehorses** | **AON/MOL** | 2.56 |  |  |  |  |  |  |  |
|  | **AON/ORB** | 1.85 | > 0.05 |  |  |  |  |  |  |
|  | **ORB/MOL** | 2.41 | > 0.05 | > 0.05 |  |  |  |  |  |
|  | **ORB/CV** | 0.47 | > 0.05 | > 0.05 | > 0.05 |  |  |  |  |
|  | **MOL/ZP** | 0.91 | > 0.05 | > 0.05 | > 0.05 |  |  |  |  |
|  | **ZP/CV** | 2.71 | > 0.05 | > 0.05 | > 0.05 | > 0.05 | > 0.05 |  |  |
|  | **ZP/CB** | -0.14 | **< 0.05** | > 0.05 | **< 0.05** | > 0.05 | > 0.05 | **< 0.05** |  |
|  | **CB/CV** | 1.16 | > 0.05 | > 0.05 | > 0.05 | > 0.05 | > 0.05 | > 0.05 | > 0.05 |
|  |  | **PLS effect size** | **AON/MOL** | **AON/ORB** | **ORB/MOL** | **ORB/CV** | **MOL/ZP** | **ZP/CV** | **ZP/CB** |
| **Mongolian horses** | **AON/MOL** | 2.55 |  |  |  |  |  |  |  |
|  | **AON/ORB** | 1.27 | > 0.05 |  |  |  |  |  |  |
|  | **ORB/MOL** | 2.17 | > 0.05 | > 0.05 |  |  |  |  |  |
|  | **ORB/CV** | 1.74 | > 0.05 | > 0.05 | > 0.05 |  |  |  |  |
|  | **MOL/ZP** | 0.23 | > 0.05 | > 0.05 | > 0.05 |  |  |  |  |
|  | **ZP/CV** | 0.88 | > 0.05 | > 0.05 | > 0.05 | > 0.05 | > 0.05 |  |  |
|  | **ZP/CB** | 0.86 | > 0.05 | > 0.05 | > 0.05 | > 0.05 | > 0.05 | > 0.05 |  |
|  | **CB/CV** | 1.44 | > 0.05 | > 0.05 | > 0.05 | > 0.05 | > 0.05 | > 0.05 | > 0.05 |
|  |  | **PLS effect size** | **AON/MOL** | **AON/ORB** | **ORB/MOL** | **ORB/CV** | **MOL/ZP** | **ZP/CV** | **ZP/CB** |
| **Przewalski's horses** | **AON/MOL** | 2.83 |  |  |  |  |  |  |  |
|  | **AON/ORB** | 0.73 | > 0.05 |  |  |  |  |  |  |
|  | **ORB/MOL** | 1.44 | > 0.05 | > 0.05 |  |  |  |  |  |
|  | **ORB/CV** | -0.71 | **< 0.05** | > 0.05 | > 0.05 |  |  |  |  |
|  | **MOL/ZP** | 1.86 | > 0.05 | > 0.05 | > 0.05 |  |  |  |  |
|  | **ZP/CV** | 1.23 | > 0.05 | > 0.05 | > 0.05 | > 0.05 | > 0.05 |  |  |
|  | **ZP/CB** | 0.50 | > 0.05 | > 0.05 | > 0.05 | > 0.05 | > 0.05 | > 0.05 |  |
|  | **CB/CV** | -0.57 | **< 0.05** | > 0.05 | > 0.05 | > 0.05 | > 0.05 | > 0.05 | > 0.05 |
